# Incidence of post-acute COVID-19 symptoms across healthcare settings in seven countries: an international retrospective cohort study using routinely-collected data

**DOI:** 10.1016/j.eclinm.2024.102903

**Published:** 2024-10-30

**Authors:** Junqing Xie, Kim López-Güell, Daniel Dedman, Talita Duarte-Salles, Raivo Kolde, Raúl López-Blasco, Álvaro Martínez, Gregoire Mercier, Alicia Abellan, Johnmary T. Arinze, Zara Cuccu, Antonella Delmestri, Dominique Delseny, Sara Khalid, Chungsoo Kim, Ji-woo Kim, Kristin Kostka, Cora Loste, Lourdes Mateu, Miguel A. Mayer, Jaime Meléndez-Cardiel, Núria Mercadé-Besora, Mees Mosseveld, Akihito Nishimura, Hedvig M.E. Nordeng, Jessie O. Oyinlola, Laura Pérez-Crespo, Marta Pineda-Moncusí, Juan Manuel Ramírez-Anguita, Nhung T.H. Trinh, Anneli Uusküla, Bernardo Valdivieso, Theresa Burkard, Edward Burn, Martí Català, Daniel Prieto-Alhambra, Roger Paredes, Annika M. Jödicke

**Affiliations:** a Nuffield Department of Orthopaedics, Rheumatology and Musculoskeletal Sciences, University of Oxford, Oxford, UK; b CPRD, Medicines and Healthcare Products Regulatory Agency, London, UK; c Fundació Institut Universitari per a la recerca a l'Atenció Primària de Salut Jordi Gol i Gurina (IDIAPJGol), Barcelona, Spain; d Department of Medical Informatics, Erasmus University Medical Center, Rotterdam, the Netherlands; e Institute of Computer Science, University of Tartu, Tartu, Estonia; f Biocomputing Unit, Aragon Health Sciences Institute (IACS), Zaragoza, Spain; g The Health Research Institute Hospital La Fe, Avenida Fernando Abril Martorell, 106 Torre A 7a Planta, 46026, Valencia, Spain; h Department of Infectious Diseases, Hospital Germans Trias i Pujol, Badalona, Catalonia, Spain; i Public Health Department, University Hospital of Montpellier, 34295 Montpellier, France; j IDESP, Université de Montpellier, INSERM, 34000, Montpellier, France; k Department of Biomedical Sciences, Ajou University Graduate School of Medicine, Suwon, Republic of Korea; l Big Data Department, Health Insurance Review and Assessment Service, Wonju, Republic of Korea; m The OHDSI Center at the Roux Institute, Northeastern University, Portland, ME, USA; n Parc de Salut Mar, Hospital del Mar Medical Research Institute, Barcelona, Spain; o Pharmacoepidemiology and Drug Safety Research Group, Department of Pharmacy, Faculty of Mathematics and Natural Sciences, University of Oslo, Oslo, Norway; p Department of Child Health and Development, Norwegian Institute of Public Health, Oslo, Norway; q Center for Global Health and Diseases, Department of Pathology, Case Western Reserve University School of Medicine, Cleveland, OH, USA; r Institute of Family Medicine and Public Health, University of Tartu, Tartu, Estonia; s Department of Biostatistics, Bloomberg School of Public Health, Johns Hopkins University, Baltimore, MD, USA; t The University and Polytechnic La Fe Hospital of Valencia, Avenida Fernando Abril Martorell, 106 Torre H 1a Planta, 46026, Valencia, Spain; u CIBER Infectious Diseases (CIBERINFEC), Institute of Health Carlos III (ISCIII), Madrid, Spain; v Universitat Autònoma de Barcelona, Catalonia, Spain; w REICOP (Red de Investigación Covid Persistente), Madrid, Spain; x Fundació Lluita Contra les Infeccions, Badalona, Catalonia, Spain; y Universitat de Vic – UCC, Vic, Catalonia, Spain; z IrsiCaixa AIDS Research Institute, Germans Trias i Pujol Research Institute (IGTP), Can Ruti Campus, Badalona, Catalonia, Spain

**Keywords:** Post-acute COVID-19 condition, Real world data, Incidence of post-acute COVID-19 symptoms, Epidemiology, International cohort study

## Abstract

**Background:**

The World Health Organisation (WHO) has identified a range of symptomatic manifestations to aid in the clinical diagnosis of post-COVID conditions, herein referred to as post-acute COVID-19 symptoms. We conducted an international network cohort study to estimate the burden of these symptoms in North American, European, and Asian populations.

**Methods:**

A federated analysis was conducted including 10 databases from the United Kingdom, Netherlands, Norway, Estonia, Spain, France, South Korea, and the United States, between September 1st 2020 and latest data availability (which varied from December 31st 2021 to February 28th 2023), covering primary and secondary care, nationwide registries, and claims data, all mapped to the Observational Medical Outcomes Partnership Common Data Model (OMOP CDM). We defined two cohorts for the main analyses: a SARS-CoV-2 infection cohort [positive polymerase chain reaction (PCR) or rapid lateral flow test (LFT) result or clinical COVID-19 diagnosis] and a general population cohort. Individuals with less than 365 days of prior history or 120 days of follow-up were excluded. We estimated incidence rates (IRs) of the 25 WHO-proposed post-acute COVID-19 symptoms, considering symptoms that occurred ≥90 and ≤365 days after index date, excluding individuals with the respective symptoms 180 days prior to the index event. Stratified analyses were conducted by age and sex. Incidence rate ratios (IRRs) were calculated comparing rates in the infected cohort versus the general population. Results from the different databases were combined using random-effects meta-analyses.

**Findings:**

3,019,408 individuals were included in the infection cohort. 1,585,160 of them were female and 1,434,248 of them male. 929,351,505 individuals were included in the general population group. 461,195,036 of them were female and 466,022,004 of them male. The 1-year IR of any post-acute COVID-19 symptom in the COVID-19 infection cohort varied significantly across databases, from 4.4 (95% CI 3.8–5.1) per 100 person-years to 103.9 (95% CI 103.2–104.7). The five most common symptoms were joint pain (from 1.6 (95% CI 1.3–1.9) to 14.3 (95% CI 14.1–14.6)), abdominal pain (from 0.3 (95% CI 0.1–0.5) to 9.9 (95% CI 9.7–10.1)), gastrointestinal issues (from 0.6 (95% CI 0.4–0.9) to 13.3 (95% CI 13.1–13.6)), cough (from 0.3 (95% CI 0.2–0.5) to 9.1 (95% CI 8.9–9.3)), and anxiety (from 0.8 (95% CI 0.6–1.2) to 11.4 (95% CI 11.2–11.6)); whereas muscle spasms (from 0.01 (95% CI 0.008–0.2) to 1.7 (95% CI 1.6–1.8)), pins and needles (from 0.05 (95% CI 0.03–0.0.9) to 1.5 (95% CI 1.4–1.6)), memory issues (from 0.03 (95% CI 0.02–0.06) to 0.8 (95% CI 0.7–0.8)), cognitive dysfunction (from 0.007 (95% CI 0.004–0.01) to 0.6 (95% CI 0.4–0.8)), and altered smell and/or taste (from 0.04 (95% CI 0.03–0.04) to 0.7 (95% CI 0.6–0.8)) were least common. Incidence rates of any post-acute COVID-19 symptoms generally increased with age, with certain symptoms peaking in middle-aged adults (anxiety, depressive disorders, headache, altered smell and taste) and others in pre-school children (gastrointestinal issues and cough). Females had higher incidence rates for most symptoms. Based on the random-effects model, the infected cohort had a higher incidence of any post-acute COVID-19 symptom than the general population, with a meta-analytic incidence rate ratio (meta-IRR) of 1.4 (1–2). A similar pattern was seen for all individual symptoms. The highest meta-IRRs were depressive disorder, 2.6 (1.7–3.9); anxiety, 2.3 (1.4–3.8); allergy, 2.1 (1.7–2.8) and sleep disorders, 2.1 (1.5–2.6). The meta-IRR for altered smell and/or taste was 1.9 (1.3–2.8).

**Interpretation:**

Post-acute COVID-19 symptoms, as listed by the WHO, were commonly observed following COVID-19 infection. However, even after standardising research methods, there was significant heterogeneity in the incidence rates from different healthcare settings and geographical locations. This is the first international study of the epidemiology of post-acute COVID-19 symptoms using the WHO-listed symptoms. Its findings contibute to understand the epidemiology of this condition from a multinational approach. Limitations of this study include the lack of consensus of the post-acute COVID-19 definition, as well as the difficulty to capture the impact on daily life of the post-acute COVID-19 symptoms in the available datasets.

**Funding:**

This work has been funded by the European Health Data Evidence Network (EHDEN) through an Evidence Generation Fund Grant and by the 10.13039/501100000272National Institute for Health and Care Research (NIHR) Oxford Biomedical Research Centre (BRC).

**Research in context**

**Evidence before this study**

We conducted a systematic literature review to characterise post-acute COVID-19 symptoms as recorded in real world data including studies up until 22 October 2021 in NIH LitCOVID, Ovid Embase, Ovid MEDLINE, Ovid Global Health, EBSCOhost CINAHL, medRxiv and WHO COVID database. Studies were reviewed if they reported persistent or new symptoms assessed at 28 days or more after onset of confirmed or clinically suspected COVID-19 disease. The 14 final studies meeting inclusion criteria showed notable heterogeneity of persistent symptoms. Likewise, previous systematic reviews, including Nalbandian et al., Lopez-Leon et al., Michelen et al., and the ECDC supported the variety of unspecific symptoms reported following recovery from acute COVID-19, highlighting the heterogenous presentation of post- COVID condition. Its reported prevalence ranged from 5% to 60% across individual studies.

At the same time, in October 2021, the World Health Organisation (WHO) published their clinical case definition of post-acute COVID-19, which included 25 different symptoms commonly reported after a SARS-CoV-2 infection that were selected through a Delphi expert consensus. Due to the high variability of the symptoms reported in the reviewed literature, we decided to use the WHO definition for our analyses.

**Added value of this study**

Using data from ten real-world databases, this is the first international study of the epidemiology of post-acute COVID-19 symptoms as defined based on the WHO criteria. Applying standardised definitions and study methodologies allowed us to compare incidence rates for post-acute COVID-19 symptoms from different healthcare settings and geographical locations and unravel the heterogeneity of post-acute COVID-19 symptoms beyond differences in their definitions.

**Implications of all the available evidence**

This study corroborates previous research of the high prevalence and heterogeneity of post-acute COVID-19 symptoms, across different geographies and healthcare settings. The differences observed between different databases reveal the complex nature of this condition and call for a multidimensional approach in understanding and managing it.

## Introduction

The COVID-19 pandemic, caused by the SARS-CoV-2 coronavirus, has significantly impacted public health worldwide.^1^ A major ongoing challenge is the post-COVID condition (PCC), also known as Long COVID.^2^ This condition is different from the acute phase of the illness. It involves a variety of symptoms that can last for months after the initial infection has cleared. Some of these symptoms might be completely new after recovery, while others could continue from the infection phase, often changing in intensity or reappearing over time.^3^

Currently, PCC remains diagnosed with patient's subjective experiences or clinician observations, as there are no objective diagnostic markers or tests available. The National Institute for Health and Care Excellence (NICE) in its ongoing review published in December 2020 outlines a timeline-based categorisation of COVID-19 symptoms, classifying those up to four weeks as acute and from four to twelve weeks as ongoing.^4^ Symptoms persisting beyond twelve weeks, in the absence of an alternate diagnosis, are considered indicative of PCC. Later, in December 2022, the World Health Organisation (WHO) adopted similar diagnostic principles and published a list of post-acute COVID-19 symptoms for the identification of patients, based on a Delphi expert consensus.^5^^,^^6^

Understanding the population burden of patients experiencing post-acute COVID-19 symptoms is crucial, and has profound implications for treatment strategies, healthcare planning, and post-pandemic management.^7^ However, estimating the epidemiology is challenging, with earlier studies reporting prevalence rates ranging from 5% to 60%.^8^ This wide variation is attributed not only to the complex and dynamic nature of post-acute COVID-19 symptoms but also to differing methodologies in research and outcome definitions. Some studies rely on routinely collected health data from electronic medical records and health claims,^9^^,^^10^ while others are based on patient self-reports.^11^^,^^12^ The temporal criterion for post-acute COVID-19 symptoms diagnosis also varies widely, with some studies considering periods as short as one-month post-infection, and others extending beyond a year.^13^ Furthermore, there is substantial inconsistency in the range of symptoms utilised, ranging from a focus on respiratory symptoms to encompassing multi-organ system involvement.^9^

In such circumstances, standardisation of study methodologies for the use of observational data is imperative to accurately characterise the epidemiology of post-acute COVID-19 symptoms. Therefore, we conducted a network study using large healthcare data from numerous regions and healthcare settings, all previously mapped to a common data model (CDM): the Observational Medical Outcomes Partnership (OMOP) CDM. We used a uniform protocol and analytical script to: (1) estimate the incidence of 25 WHO-defined post-acute COVID-19 symptoms following COVID-19, (2) assess the demographic patterns of these symptoms, and (3) compare their incidence among the infected, test-negative, and general population.

## Methods

This study is reported following the STROBE guidelines for cohort studies.^14^

### Data sources

We used routinely collected, de-identified healthcare datasets from different countries and healthcare settings.

We retrieved primary care electronic health records of people registered with their general practitioners from the Clinical Practice Research Datalink (CPRD GOLD and Aurum, UK),^15^^,^^16^ the Integrated Primary Care Information database (IPCI, The Netherlands)^17^ and the Information System for the Development of Research in Primary Care (SIDIAP, Spain).^18^

We used hospital electronic health records from the Parc de Salur Mar Barcelona Information System (IMASIS, Spain),^19^ the Centre Hospitalier Universitaire de Montpellier (eDOL, France)^20^ and the Ajou University School of Medicine (AUSOM, South Korea).

We also retrieved adjudicated medical and pharmacy claims from the US (PharMetrics Plus for Academics (PharMetrics, US),^21^ national health insurance claims from Estonia (CORIVA, Estonia),^22^ as well as linked nationwide healthcare data covering both primary and secondary care from Norway (NLHR@UiO Norway).^23^

All databases contained information on people's demographics and clinical information in the form of coded diagnoses, symptoms and prescriptions collected from GP practices, hospitals or health claims.

All databases were previously mapped to the Observational Medical Outcomes Partnership (OMOP) Common Data Model.^24^ This allowed us to perform the federated network study without sharing patient-level data.

### Data statements

Use of Clinical Practice Research Datalink (CPRD) data for this study was approved via the Research Data Governance (RDG) Process of the UK Medicines and Healthcare Products Regulatory Agency (protocol 23_002603).

Ethical approval for NHR@UiO in this study was obtained from The Regional Committee for Research Ethics (approval number 155294) and the Data Protection Officer at the University of Oslo (approval number 523275).

Ethical approval for CORIVA data was obtained from the Research Ethics Committee of the University of Tartu (No. 351/M-8). Ethical approval for IMASIS was obtained by the Parc de Salut Mar Research Ethics Committee CEIm-Parc de Salut Mar (number 2021/9975).

Ethical approval for IPCI was obtained by the Integrated Primary Care Information review board (registration number 9/2023).

For eDOL, no ethical approval was required according to French law for this study. All patients admitted to the hospital are provided with general information about the collection and secondary use of their data, and an opt-out option is offered.

PharMetrics® Plus for Academics needed no approval for use of pseudoanonymised secondary data.

This study was approved by the institutional review board of Ajou University Medical Center (AJOUIRB-MDB-2021-694).

For SIDIAP, ethical approval was obtained by the IDIAP Jordi Gol Research Ethics Committee CEIm (number 22/177-PCV).

### Study cohorts

We included all individuals registered in the participating databases and with a recorded COVID-19 diagnosis (confirmed or suspected) or positive SARS-CoV-2 test (polymerase chain reaction (PCR) or rapid lateral flow test (LFT)) after 01/09/2020. We excluded people with COVID-19 recorded before study start and with influenza in the 90 days before the SARS-CoV-2 infection. People with less than 365 days of prior history and follow-up of less than 120 days were also excluded. Individuals’ follow-up was censored at 365 days, on the end of their observation, death or end of COVID-19 testing in their country if applicable.

We curated a general population cohort by including all participants registered in the database at 01/09/2020, with the same eligibility criteria applied as the infection cohort. We also created a test-negative cohort by using the same criteria as above but substituting the SARS-Cov-2 positive test or COVID-19 diagnosis by a SARS-Cov-2 negative test.

All the data used for this study were secondary health data recorded during routine healthcare encounters and pseudonymised for research.

### Outcomes

For the post-acute COVID-19 symptoms, we used the list of 25 symptoms published in the WHO clinical case definition for “post COVID-19” condition, including abdominal pain, allergy, altered smell and/or taste, anxiety, blurred vision, chest pain, cognitive dysfunction, cough, depression, dizziness, dyspnoea, fatigue or malaise, gastrointestinal issues (acid reflux, constipation, or diarrhoea), headache, intermittent fever, joint pain, memory issues, menstrual problems, muscles spasms or pain, neuralgia, pins and needles sensation, sleep disorder, tachycardia, post-exertional fatigue and tinnitus and hearing problems.^6^ Code lists were developed separately for each symptom and reviewed independently by 3 clinicians. We also studied the additional outcome of having any of the symptoms.

### Statistical analyses

We calculated the incidence rates of the post-acute COVID-19 symptoms on the infection cohort and the general population for the main analysis.

For the general population, an individual started contributing time to the denominator if they had at least 365 days of previous history in the dataset. The incidence of all the post-acute COVID-19 symptoms was calculated for that population in the different time intervals of interest (yearly and monthly from the study start date to the latest data availability for each dataset).

For the infected population, individuals started contributing to the denominator when they had a record or diagnosis of a COVID-19 infection. The incidence of all the post-acute COVID-19 symptoms was calculated, provided that the symptom happened in the window of 90–365 days after index event (the positive COVID-19 test or diagnosis). This way we neglected the acute phase from 1 to 90 days after index event for the calculations. Moreover, we demanded no record of the symptom in the 180 previous days before index event to minimise misclassification of pre-existing, chronic symptoms, such as anxiety or depression.

For the two denominators (general population and infected cohort), we did not allow repeated events. This means that once an individual, according to our definition, had a positive record of a post-acute COVID-19 symptom, they stopped contributing to the denominator of eligible population. Furthermore, we stratified all analyses by age groups in years (0,6), (7,11), (12,18), (19,40), (41,64), (65+) and by sex (male and female).

Records of demographic covariates used for age and sex stratification were available for all individuals, with no missingness. The absence of a record of a symptom was considered the absence of that symptom.

Crude incidence rate ratios and 95% confidence intervals were calculated by comparing the incidence rates of any and each of the 25 symptoms in the infection cohort with that in the comparator cohort (general population) without adjustment of any potential confounders. The ratios across databases were finally pooled using the random-effects model. The meta-analysis was performed using the package meta in R, with the inverse variance method.^25^

We mainly used the package IncidencePrevalence for the incidence calculations.^26^ All the analytical code can be found in a GitHub repository for transparency: https://github.com/oxford-pharmacoepi/LongCovidStudyathon_W1/tree/main.

Details on the methods used for the sensitivity analyses using the test-negative cohort can be found in the Supplementary Materials.

### Role of the funding source

The funder of the study had no role in study design, data collection, data analysis, data interpretation, or writing of the manuscript. Information on data access for all databases can be found in the Contributors section at the end of the manuscript. JX, DPA and AMJ were responsible for the decision to submit for publication.

## Results

### Characteristics of study databases

Table 1 depicts the key characteristics of all participating databases. There were two individual databases from the UK and Spain, with the remaining six from six different countries. They covered healthcare settings of primary care, secondary care, nationwide registry, and claims. The largest source populations were from the PharMetrics® Plus for Academics database (107 million historical and active people), followed by CPRD Aurum (40 million historical and active people), CPRD GOLD (21 million historical and active people), and SIDIAP (6 million historical and active people).

**Table 1:** Overview of participating databases.

Database name	Acronym	Country	Healthcare setting	Number of people	Study end	Infection cohort	Test negative cohort
Clinical Practice Research Datalink GOLD	CPRD GOLD	United Kingdom	Primary care	21 million	06/2022	Available	Available
Clinical Practice Research Datalink Aurum	CPRD Aurum	United Kingdom	Primary care	40 million	03/2021	Available	Available
The Information System for Research in Primary Care	SIDIAP	Spain	Primary care	6 million	06/2022	Available	NA
The Integrated Primary Care Information	IPCI	The Netherlands	Primary care	3 million	12/2022	Available	Available
Norwegian linked health registry data	NLHR@UiO	Norway	Nationwide registry	5.4 million	12/2021	Available	NA
Hospital records from Parc Salut Mar Barcelona	IMASIS	Spain	Secondary care	2 million	20/2022	Available	Available
The Centre Hospitalier Universitaire de Montpellier	eDOL	France	Secondary care	2 million	12/2022	Available	Available
Hospital records from Ajou University Medical Centre	AUSOM	South Korea	Secondary care	2.7 million	02/2023	Available	Available
Healthcare claims from Estonia	CORIVA	Estonia	Claims	300K	02/2022	Available	Available
PharMetrics® Plus for Academics	PharMetrics Plus	US	Claims	107 million	06/2022	Available	NA

There were no records of “post-extersional fatigue” in any of the databases, so we have removed this symptom from the plots.

### Incidence of post-acute COVID-19 symptoms across the databases

**Fig. 1:**
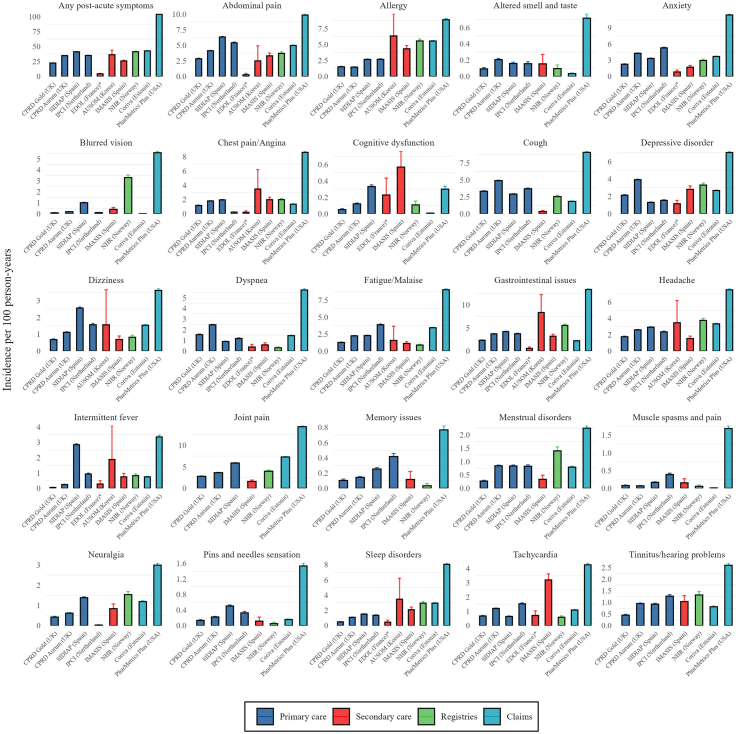
**Incidence rate of post-acute COVID-19 symptoms in the infected cohort**. Note: Plots have been scaled independently so that all incidences can be visualised.

The 1-year incidence rates (IR) of any post-acute COVID-19 symptoms were high and varied across databases. In primary care databases, IRs ranged from 22.5 (95% CI 22.2–22.7) per 100 person-years in CPRD GOLD (UK) to 41.3 (95% CI 41.0–41.5) in SIDIAP (Spain) (Fig. 1). Secondary care databases reported IRs from 4.4 (95% CI 3.8–5.1) in eDOL (France) to 36.3 (95% CI 29.7–44.0) in AUSOM (South Korea). Notably, registry and claims databases recorded higher incidences, with the NLHR@UiO (Norway) registry reporting 41.3 (95% CI 40.5–42.2) and claims databases showing IRs from 42.8 (95% CI 42.6–43.0) in CORIVA (Estonia) to 103.9 (95% CI 103.2–104.7) in PharMetrics Plus (US).

Among individual symptoms, the five most common were joint pain (IR range: 1.6 [95% CI 1.3–1.9] to 14.3 [95% CI 14.1–14.6]), abdominal pain (0.3 [95% CI 0.1–0.5] to 9.9 [95% CI 9.7–10.1]), gastrointestinal issues (0.6 [95% CI 0.4–0.9] to 13.3 [95% CI 13.1–13.6]), cough (0.3 [95% CI 0.2–0.5] to 9.1 [95% CI 8.9–9.3]), and anxiety (0.8 [95% CI 0.6–1.2] to 11.4 [95% CI 11.2–11.6]). In contrast, muscle spasms and pain, pins and needles, memory issues, cognitive dysfunction, and altered smell and/or taste were among the least frequently recorded symptoms, with IRs below 1 per 100 person-years in most databases.

Patients treated in secondary care for their initial COVID-19 generally reported higher incidences of a few specific symptoms, including gastrointestinal issues, allergies, sleep disorders, tachycardia, and cognitive dysfunction compared to primary care data. Nationwide registry data closely aligned with primary care databases for most individual symptoms. Importantly, PharMetrics Plus claims data consistently showed the highest incidence rates for all symptoms across all databases.

The 1-year incidence rate (IR) of all post-acute COVID-19 symptoms for the general population and the test negative cohorts can be found in the Supplementary Materials (Figures S1 and S2).

### Rates of post-acute COVID-19 symptoms stratified by age and sex

**Fig. 2:**
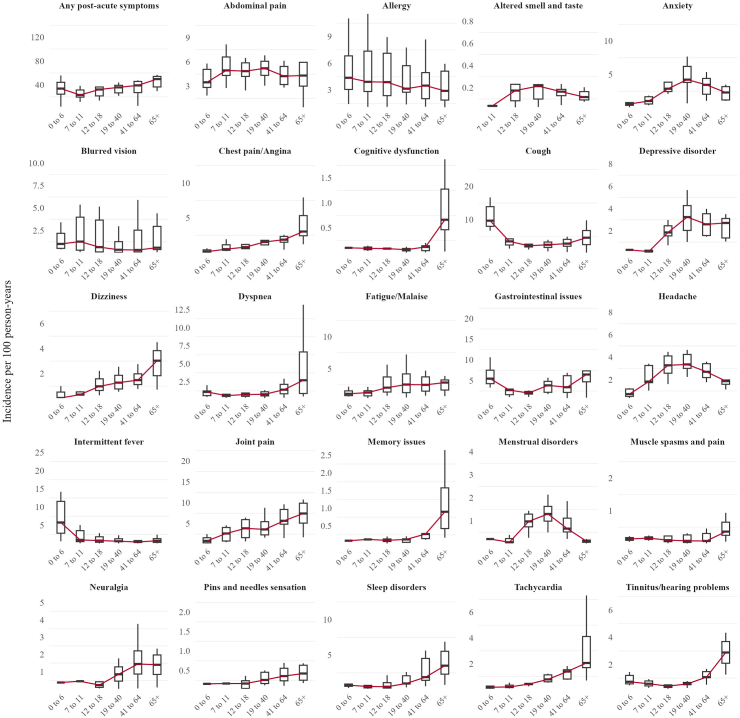
**Distribution of incidence rate of post-acute COVID-19 symptoms by age in the infected cohort**. Note: The red line represents the median of the incidences.

**Fig. 3:**
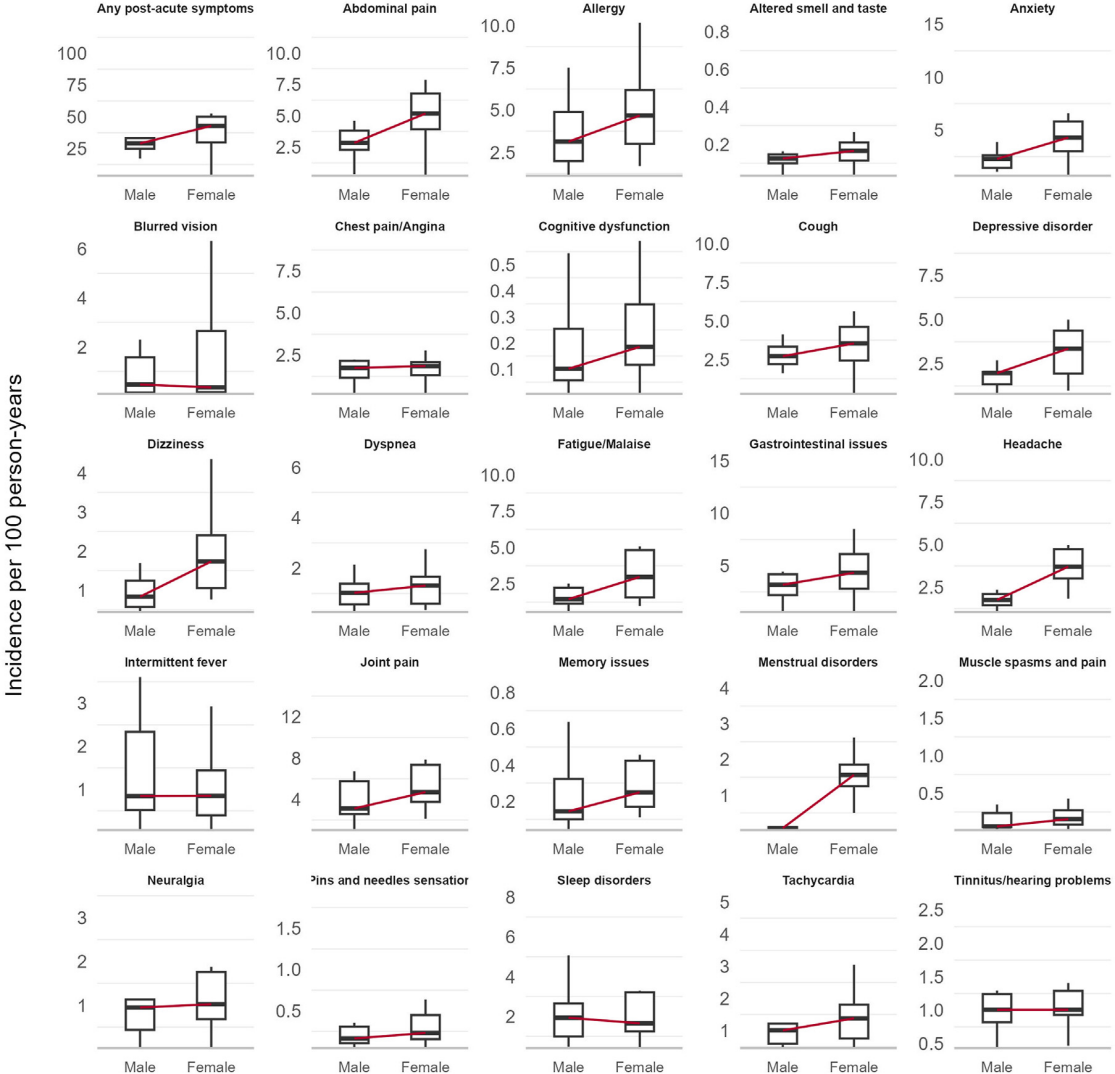
**Distribution of incidence rate of post-acute COVID-19 symptoms by sex in the infected cohort**. Note: There were very few menstrual disorder events in Male recorded in the PharMetrics Plus and SIDIAP databases. The red line represents the median of the incidences.

Figs. 2 and 3 show the pooled values of the incidence rates on the infected cohort across databases for the different post-acute COVID-19 symptoms, age and sex groups. The incidence of any post-acute COVID-19 symptoms generally increased with age (Fig. 2). The lowest median rate across all databases was observed in school-aged children (7–11 years), with an IR of 23.0 per 100 person-years, while the highest rate was in older adults (≥65 years) at 49.7. Preschool children (0–6 years) and young adults (19–40 years) had similar post-acute COVID-19 symptoms rates, with median IRs of 33.7 and 35.8 respectively.

The trend in the incidence of most individual symptoms followed that of post-acute COVID-19 symptoms overall, albeit with notable exceptions. Specifically, anxiety, headache, depressive disorders, menstrual disorders, and altered smell and/or taste displayed a reverse U-shaped pattern, peaking in middle-aged individuals. Symptoms such as gastrointestinal issues and cough showed a U-shaped distribution, with both children and older adults experiencing the highest rates.

Women experienced a higher median incidence of post-acute COVID-19 symptoms compared to men (Fig. 3). This pattern was also seen for most individual symptoms: joint pain, abdominal pain, gastrointestinal issues, cough, anxiety, allergy, fatigue, headache, depressive disorders, dizziness, menstrual disorders, memory issues, and cognitive dysfunction. Other symptoms had similar rates across sexes, with no symptom consistently more common in men.

Figures S3 and S4 in the Supplementary Materials show the pooled values of the incidence rates on the test negative cohort and general population of the post-acute COVID-19 symptoms across databases stratified by sex and age. Figures S5 and S6 show the pooled values for the infected cohort stratified by healthcare setting of the database.

### Crude rate ratio of the post-acute COVID-19 symptoms

**Fig. 4:**
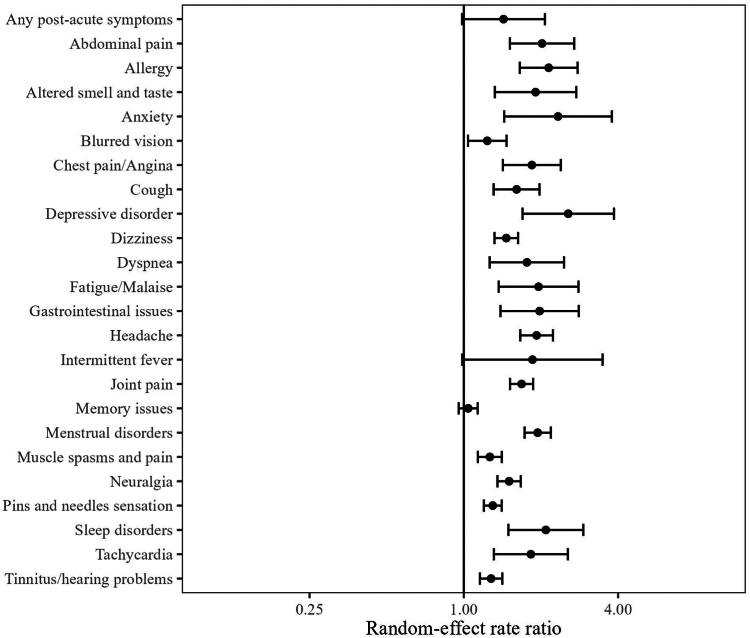
**Crude incidence rate ratio of post-acute COVID-19 symptoms between the infected cohort and general population**.

Based on the random-effects model, the infected cohort had a higher incidence of any post-acute COVID-19 symptom than the general population, with a meta-analytic incidence rate ratio (meta-IRR) of 1.4 (1–2) (Fig. 4). A similar pattern was seen for all individual symptoms. The highest meta-IRRs were depressive disorder, 2.6 (1.7–3.9); anxiety, 2.3 (1.4–3.8); allergy, 2.1 (1.7–2.8) and sleep disorders, 2.1 (1.5–2.6). The meta-IRR for altered smell and/or taste was 1.9 (1.3–2.8).

Conversely, the meta-IRR between the infection and test-negative cohort was 0.9 (95% CI 0.6–1.2) for any post-acute COVID-19 symptom. Similarly, the meta-IRR was less than 1 for most individual symptoms, indicating a lower incidence in the infected than the test-negative cohort. A few exceptions were seen for symptoms similarly common in both cohorts, including fatigue, intermittent fever and menstrual disorders. Finally, altered smell and taste was the only symptom consistently more common in the infected cohort, with meta-IRR 2.2 (1.1–4.5). These results are presented in Figure S11.

Stratified analyses by healthcare setting yielded similar results and are available in the Supplementary Materials, in Figures S7–S10.

## Discussion

This is the first international and transcontinental study of the epidemiology of post-acute COVID-19 symptoms as defined based on the WHO criteria via leveraging a network cohort analysis of ten real-world databases and has provided critical benchmarking data on the descriptive epidemiology of post-acute COVID-19 symptoms following post-acute SARS-CoV-2 infection. We observed a notably high one-year post-infection rate of any post-acute COVID-19 symptoms, which varied significantly across geographies and healthcare settings. In primary care databases, the most common post-acute COVID-19 symptoms included abdominal pain, cough, anxiety, joint pain, and fatigue. Neurological and ENT-related symptoms such as pins and needles sensations, neuralgia, and cognitive dysfunction were less frequent. Together, our results show substantial heterogeneity in the manifestation of symptoms post-acute COVID-19 infection.

Our data also showed consistently higher rates of post-acute COVID-19 symptoms (overall as well as individual symptoms) in females across all databases. Furthermore, school-aged children (7–11 years) and older adults (≥65 years) generally presented higher IRs than other age groups, albeit with variations in specific symptoms. This suggests a broader impact of post-acute COVID-19 symptoms across subpopulations, with notable vulnerabilities in females and both the very young and the elderly.

Compared to the test-negative cohort, the infected cohort had a crude lower incidence of most symptoms, but higher than in the general population. However, the incidence of altered smell and taste was notably higher in the infected cohort compared to the two controls.

Our findings corroborate previous research on the high prevalence of post-acute COVID-19 symptoms in COVID-19 survivors. A recent meta-analysis including 194 studies and 735,006 participants indicated that approximately 45% of COVID-19 survivors experienced unresolved symptoms at around four months post-infection.^27^ Our study generally aligns with this finding, showing a one-year incidence rate of any post-acute COVID-19 symptom ranging from 20 to 30 per 100 person-years in primary and secondary care databases, respectively. Notably, our estimates of incidence were substantially higher in claims and nationwide registries. This suggests that such databases may capture a higher proportion of individuals with persistent COVID-19 symptoms, possibly because transient or mild symptoms may be unrecorded in primary and secondary care settings, especially if they are not the primary reason for healthcare visits. For example, a federated analysis of 58 million patient records in England concluded that the recording of long COVID in primary care is very low and call for increased awareness of diagnostic codes.^28^ Despite this, our results collectively demonstrated the widespread occurrence of post-acute COVID-19 symptoms, whether as new or continued symptoms, across healthcare practices.

For the individual symptoms, general pains, metal health disorders and fatigue emerged to be most highly incident symptoms post-COVID-19, in line with prior epidemiologic data on this topic.^8^^,^^27^ Intriguingly, we observed a lower incidence of these symptoms in secondary care compared to primary care patients. This difference may be attributed to several factors related to the nature of healthcare settings and symptom reporting. Primary care often serves as the initial contact point for patients with general symptoms.^29^ Research indicates that fatigue accounts for 10–20% of primary care consultations. In contrast, fatigue in secondary care settings, particularly in acute care and ambulatory settings, may go unrecognised and untreated, often overshadowed by more acute or severe conditions.^30^

Our study also highlights clear sex differences in the post-acute phase of COVID-19. While it has been widely reported that men were more likely to be hospitalised or progressing during the acute illness, women, instead, were more likely to experience persistent symptoms post-COVID. This aligns with previous survey studies^31^^,^^32^ and emphasises the need for sex-specific preventive and therapeutic strategies. Additionally, our findings confirm the correlation of age with post-acute COVID-19 symptoms, showing higher incidence rates in older adults. However, this phenomenon was not uniform across all symptoms. Furthermore, consistent with previous research focusing on primary care databases in the UK and Spain, ‘altered smell and/or taste’ was significantly more frequent among COVID-19 patients compared to test-negative controls.^33^ This relationship was further validated in secondary care and claims-based databases in the current study. Moreover, our study also showed that the incidence of most symptoms was notably higher in the cohort of infected people compared to the general population. This supports the significant additional health burden associated with COVID-19. Similar results were also reported in a prior study^34^ and reflect the complexity of impacts of the pandemic at large on population health. It also highlights the crucial need for careful selection of control groups in future epidemiological research. The higher incidence of symptoms in the test-negative cohort may result from differences in healthcare seeking behaviour or other viral infections mimicking COVID-19 symptoms, or clinically more vulnerable groups. It however also shows that not all symptoms included in the WHO definition are differential for PCC. All these multifaceted nature of health outcomes during the pandemic should be considered in using real-world data.

A key strength of our study is the use of data harmonisation, crucial for improving the clinical utility and comparability of research on post-acute COVID-19. By using a common data model and shared conventions, our study ensures consistent and most granular representation of clinical information across diverse healthcare systems, making it the largest of its kind to date. Also, our approach utilised a federated analysis network for evidence synthesis. This ensured uniformity in critical study elements like inclusion and exclusion criteria, washout periods, and follow-up windows across all contributing databases and is unlike previous meta-analyses reliant on summarised statistics with considerable heterogeneity. Aligning with WHO recommendations, we predefined a specific list of symptoms rather than the broad range reported in the literature, improving the specificity in identifying post-acute COVID-19 with symptomatic data. However, our symptoms-based estimates should be interpreted cautiously as the incidence of post-acute COVID-19 condition, given that many of the studied post-acute COVID-19 symptoms are also common in the general population as shown in our study.

There are however several limitations. First, our study adopts the 90-day post-infection onset as the threshold. Although in line with common practices in literature, this definition still lacks a consensus, particularly regarding the time component of continuing symptoms. Different thresholds might produce significantly varied incidence rates or ratios. Moreover, the WHO's clinical case definition states that post-acute COVID-19 symptoms typically impact people's everyday functioning. While the impact on daily life cannot be reliably assessed based on electronic health records, people's symptoms recorded in our data were however severe enough for them to seek medical attention e.g. attended their GP. Second, the study relies on secondary use of passively collected data. Since most symptoms are subjective experiences, the symptoms recorded in the different healthcare settings are expected to vary, and under-reporting of e.g. less severe symptoms in secondary care settings is expected. Third, we did not investigate the dynamics and trajectory of symptoms post-COVID-19. Using a single symptom over a time frame may oversimplify the condition due to the potential fluctuating course. Lastly, although we included test-negative and general database population groups as controls, distinguishing the most specific post-acute symptoms following COVID-19 remains challenging. This is due to the study's design not accounting for other influential factors, such as patient comorbidities and vaccination status, and not conducting any analytic matching for the cohorts, all of which could affect the results.

In conclusion, this study provides important epidemiological data into the diverse symptomatology of post-acute COVID-19. The observed differences in symptom incidence across geographies, healthcare settings, and socio-demographics highlight the intricate nature of post-acute COVID-19 research and call for a multidimensional approach in understanding and managing this condition.

## Contributors

K.L, M.C, D.P.A, J.X and A.M.J. led the conceptualisation of the study.

K.K., D.P.A. and A.M.J. led the phenotyping of long COVID symptoms.

A.D. mapped and curated CPRD data.

K.L. and M.C. wrote the analytical code.

K.L., M.C., D.Ded., R.K., A.M., M.M., Z.C., D.Del., C.K., J.K., J.M., N.M., J.M.R., N.TH.T. conducted the statistical analyses on the respective databases.

D.Ded., T.D., R.L., G.M., A.A., J.T.A., T.B., E.B., S.K., K.K., C.L., M.A.M., A.N., H.ME.N., J.O.O., R.P., L.P.C., A.U., B.V., D.P.A., J.X., L.M. and A.M.J. clinically interpreted the results.

J.X, K.L. and A.M.J. wrote the first draft of the paper.

All authors read, contributed to, and approved the last version of the paper.

D.P.A and A.M.J. obtained the funding for this project.

N.M. and T.D. had access to and verified the Information System for Research in Primary Care (SIDIAP) data; K.L and M.C. had access to and verified the Clinical Practice Research Datalink (CPRD GOLD) data, D.Ded. and Z.C. had access to and verified the Clinical Practice Research Datalink (CPRD Aurum) data, R.K. and A.U. had access to and verified the CORIVA data; N.T and H.ME.N had access to and verified the Norwegian linked health registry data (NLHR@UiO). M.A.M and J.M.R had access to and verified the Hospital records from Parc Salut Mar Barcelona (IMASIS). M.M and J.T.A. had access to and verified the Integrated Primary Care Information database (IPCI). D.D. and G.M had access to and verified the database from the Centre Hospitalier Universitarie de Montpellier (eDOL). K.L and M.C had access to and verified the database Pharmetrics® Plus for Academics. J.K and C.K had access to and verified the Hospital records from Ajou University Medical Center (AUSOM).

J.X., D.P.A. and A.M.J. were responsible for the decision to submit for publication.

## Data sharing statement

These data are not publicly available as direct data sharing is not allowed.

## Declaration of interests

D.P.A.‘s department has received grant/s from Amgen, Chiesi–Taylor, Lilly, Janssen, Novartis, and UCB Biopharma. His research group has received consultancy fees from Astra Zeneca and UCB Biopharma. Amgen, Astellas, Janssen, Synapse Management Partners and UCB Biopharma have funded or supported training programmes organised by DPA's department. R.P. reports serving on advisory boards for Gilead Sciences, Inc, Pfizer, Inc, Roche Therapeutics, MSD, GSK, ViiV Healthcare, Eli Lilly and Company, PharmaMar, and Atea Pharmaceuticals, Inc; and receiving research grants paid to his institution from MSD, ViiV Healthcare, Gilead Sciences, and PharmaMar. L.M. reports receiving grants from Grifols; receiving honoraria as a speaker from AstraZeneca, Gilead Sciences, GSK, and Pfizer; and participation in advisory boards for Gilead Sciences and Merck. M.M. works for a research group that in the past 3 years received unconditional research grants from Chiesi, UCB, Amgen, Johnson & Johnson, Innovative Medicines Initiative and the European Medicines Agency. D.Ded., Z.C. and J.O. are employees of the Medicines and Healthcare Products Regulatory Agency, which provides the CPRD research service. K.K. is a consortial author in the US National Institutes of Health National COVID Cohort Collaborative (funding expired in 2022 with no renewal or active impact on any current work). G.M. reports receiving consulting fees from Pfizer. A.N. reports grants or contracts from Alfred P. Sloan Foundation, National Institutes of Health and the U.S. Food and Drug Administration. L.P. was supported by a Sara Borrell fellowship awarded by the Spanish Institute of Health Carlos III (CD23/00223). K.L.G is funded through an MRC scholarship with Bayer AG as an industrial partner.

All other co-authors declare no competing interests.
