# Supplementary material for: Incidence of post-acute COVID-19 symptoms across healthcare settings in seven countries: an international retrospective cohort study using routinely-collected data
Source: eClinicalMedicine. 2024 Oct 30;77:102903. doi: 10.1016/j.eclinm.2024.102903 (PMC11564986; doi:10.1016/j.eclinm.2024.102903)
Supplement: Supplementary materials [file mmc1.docx]

**Incidence of post-acute COVID-19 symptoms across healthcare settings: an international cohort study using routinely-collected data from 7 countries**

**Supplementary Materials**

**Supplementary Text**

**Methods – Test-negative cohort**

The test-negative cohort was created by using the same criteria as the infected cohort but substituting the SARS-Cov-2 positive test or COVID-19 diagnosis by a SARS-Cov-2 negative test. The rest of inclusion criteria were not changed. Only the first negative test per patient was used. Follow-up was also not changed but for censoring at a SARS-Cov-2 infection or clinical diagnosis of COVID-19.

The incidence of the post-acute COVID-19 symptoms was calculated similarly to the incidence in the infected cohort. Individuals started contributing time to the denominator at the record of the negative SARS-Cov-2 test. The windows for the outcomes, washout and other analysis parameters were identical.

Crude incidence rate ratios and 95% confidence intervals were calculated comparing the infected and test-negative cohort using the same procedure as with the general population.


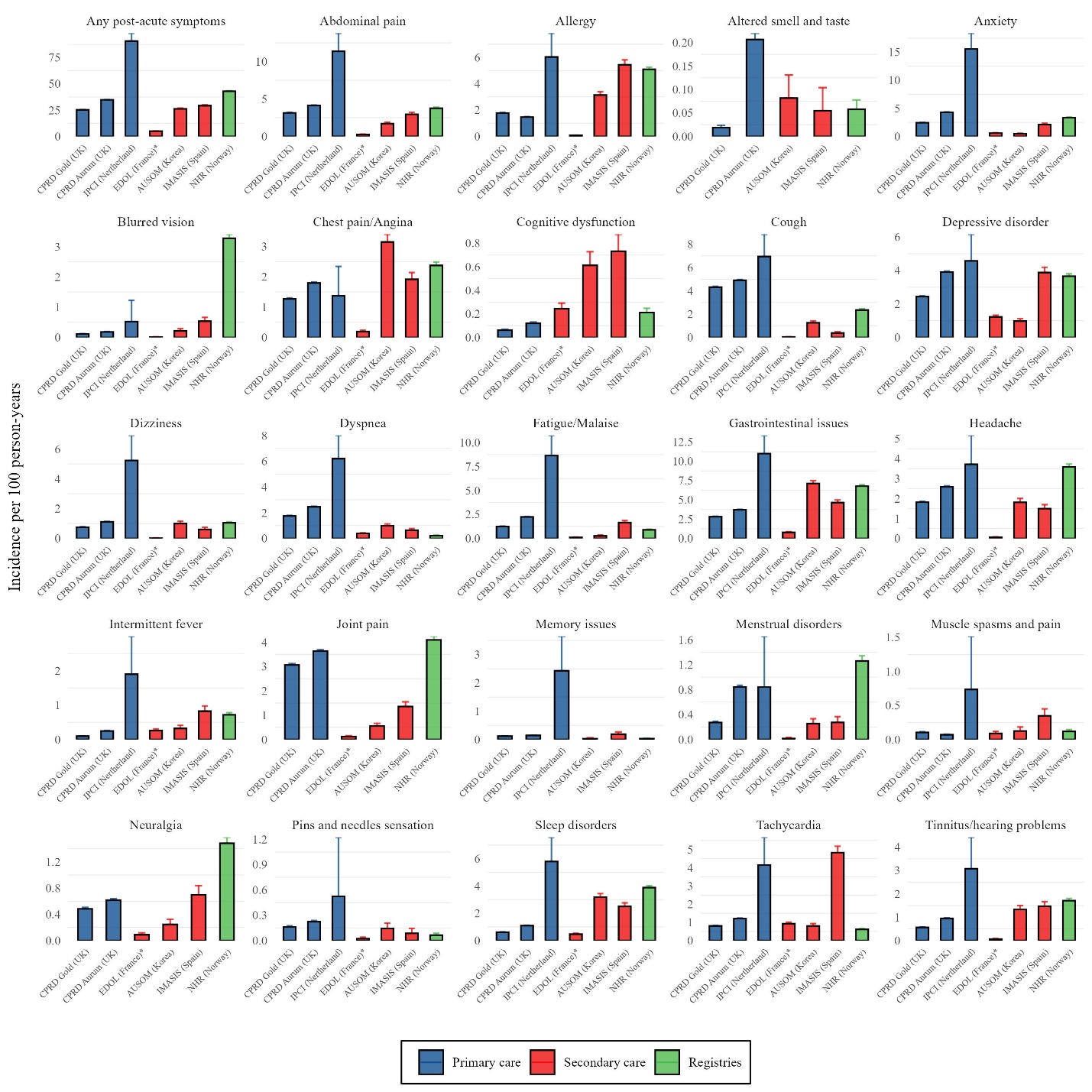


## **Figure S1 Incidence rate of post-acute COVID-19 symptoms in the test negative cohort.**

Note: Plots have been scaled independently so that all incidences can be visualised.


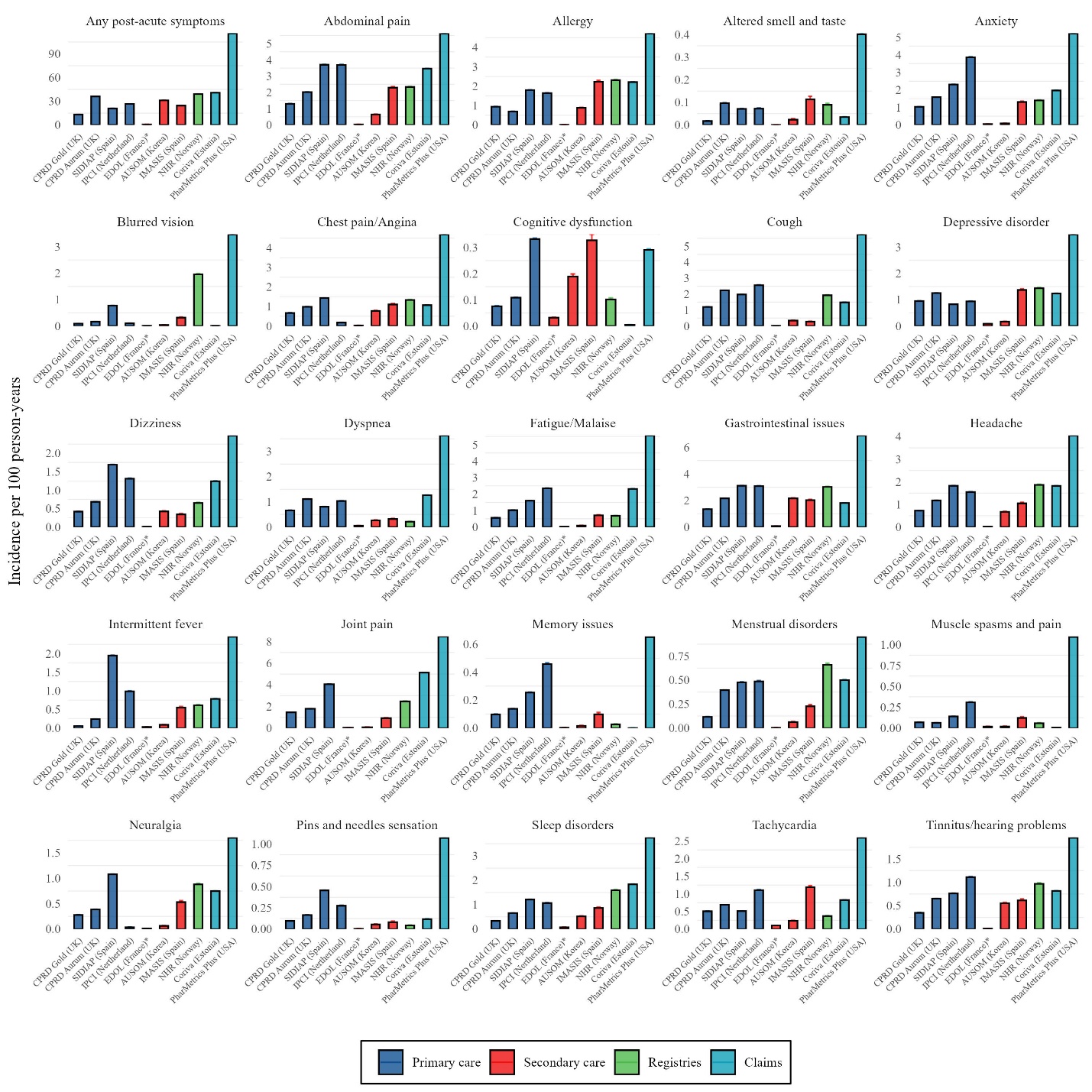


## **Figure S2 Incidence rate of post-acute COVID-19 symptoms in the general population.**

Note: Plots have been scaled independently so that all incidences can be visualised.


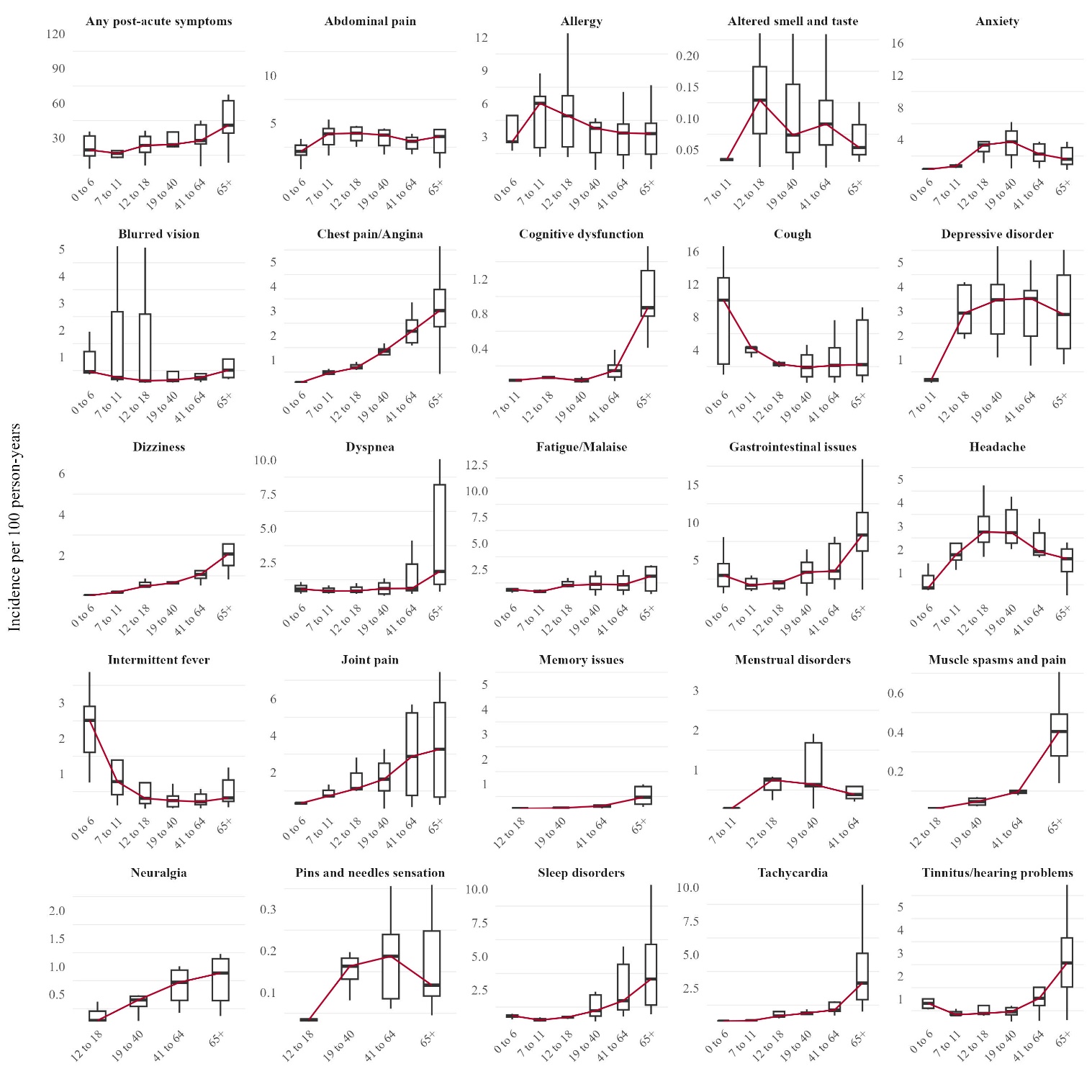


## **Figure S3 Distribution of incidence rate of post-acute COVID-19 symptoms by age in the test negative cohort.**

Note: There were very few menstrual disorder events in Male recorded in the PharMetrics Plus and SIDIAP databases.


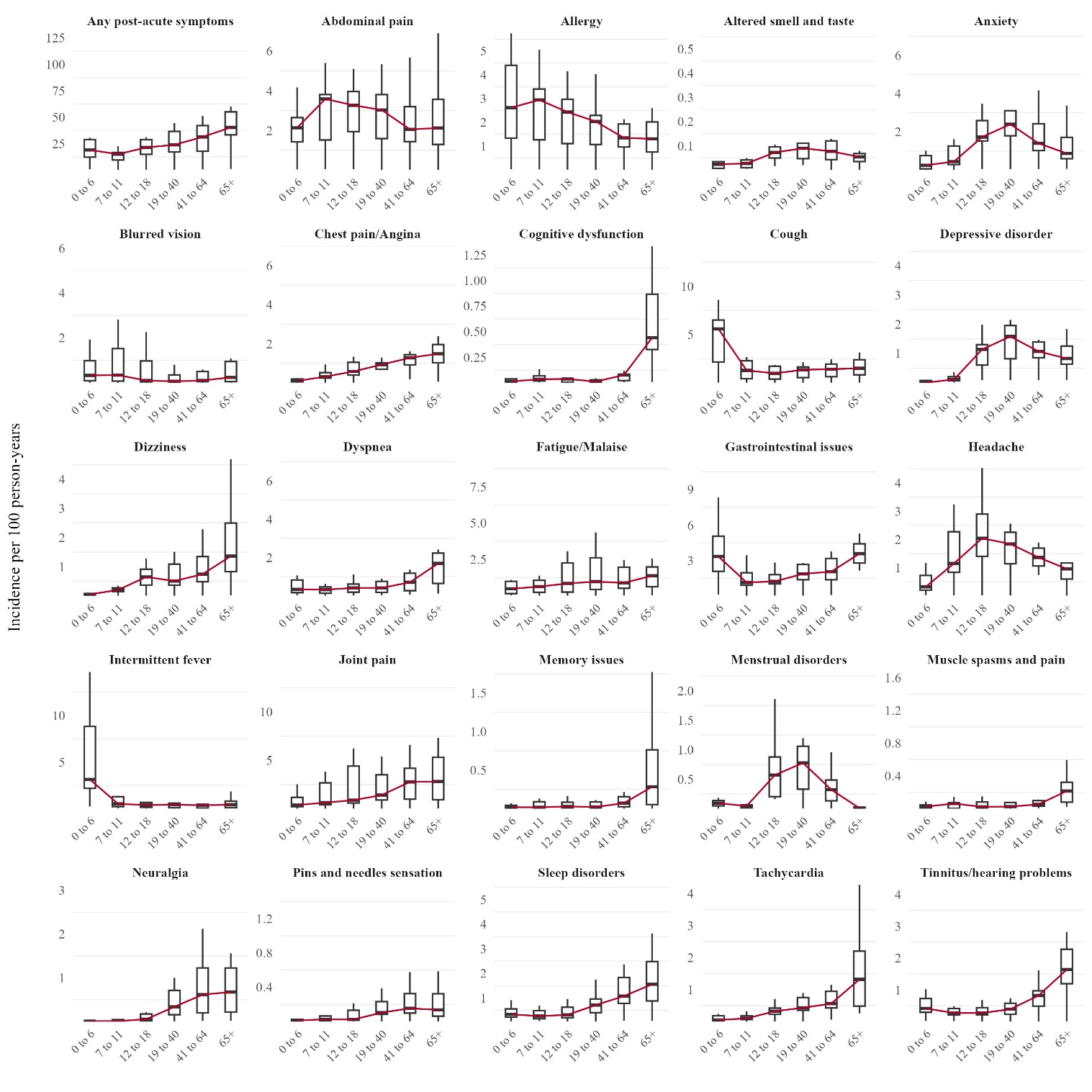


## **Figure S4 Distribution of incidence rate of post-acute COVID-19 symptoms by age in the general population.**

Note: There were very few menstrual disorder events in Male recorded in the PharMetrics Plus and SIDIAP databases.


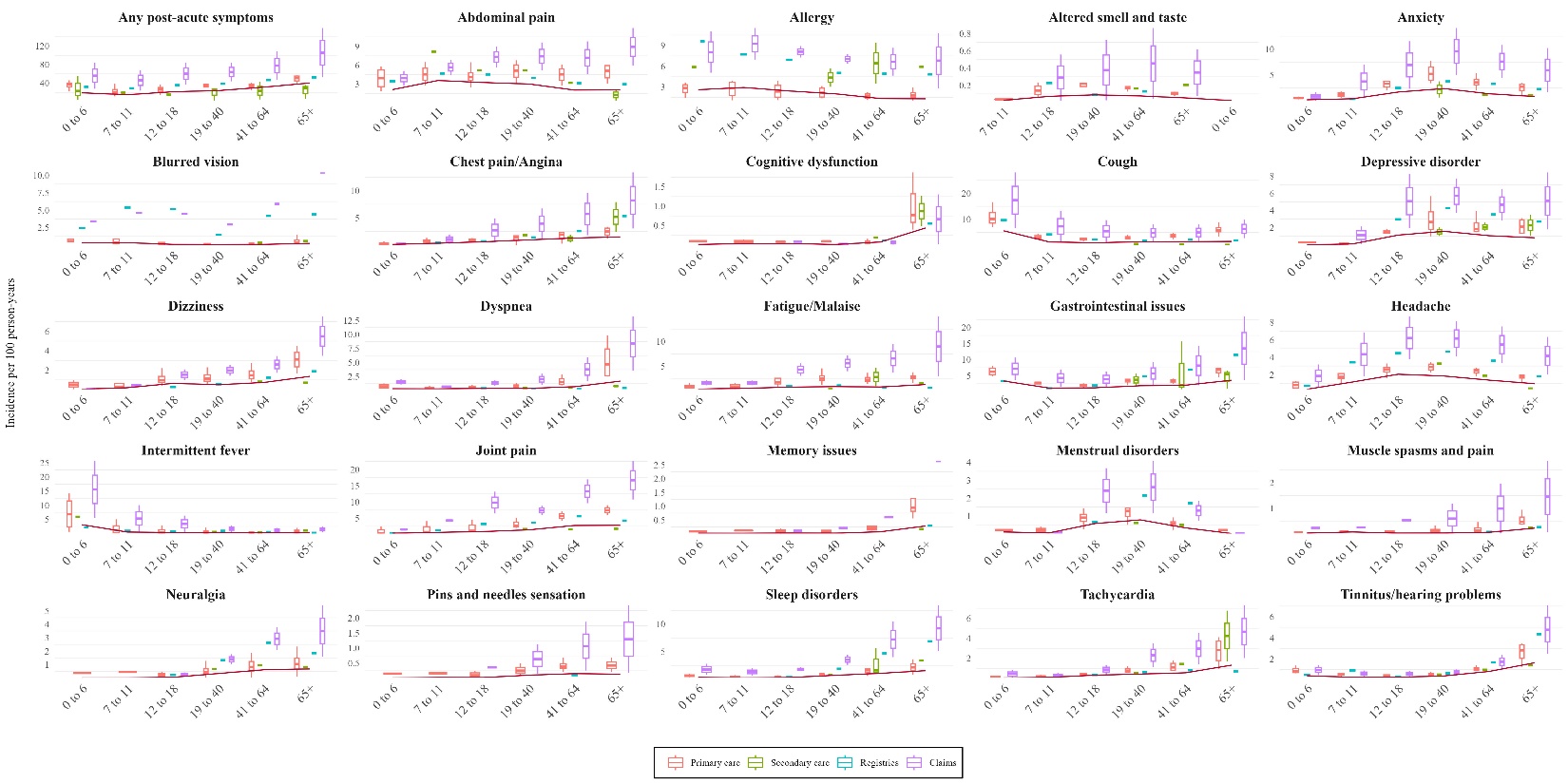


## **Figure S5 Distribution of incidence rate of post-acute COVID-19 symptoms by age in the infected cohort stratified by healthcare setting.**


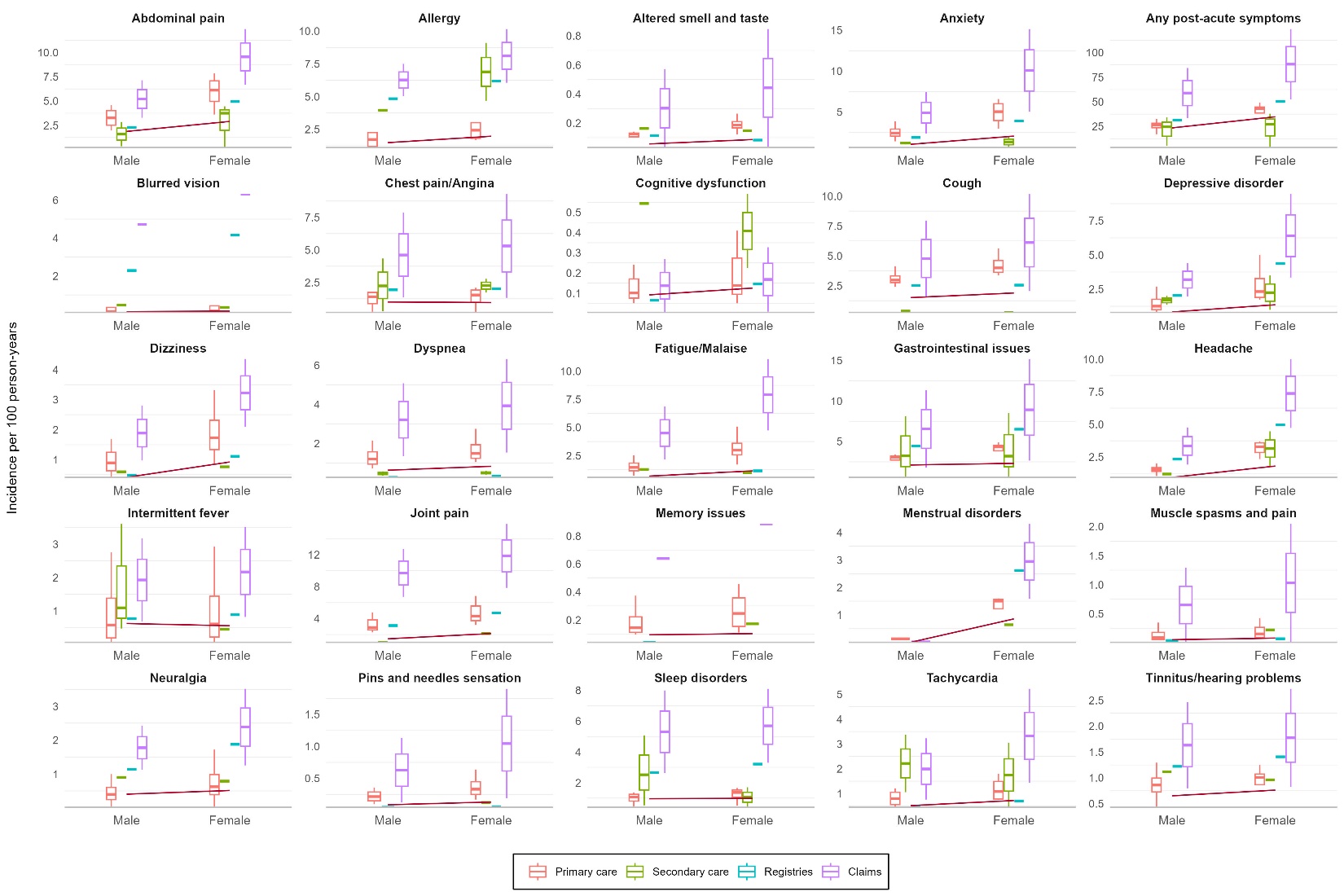


## **Figure S6 Distribution of incidence rate of post-acute COVID-19 symptoms by sex in the infected cohort stratified by healthcare setting.**


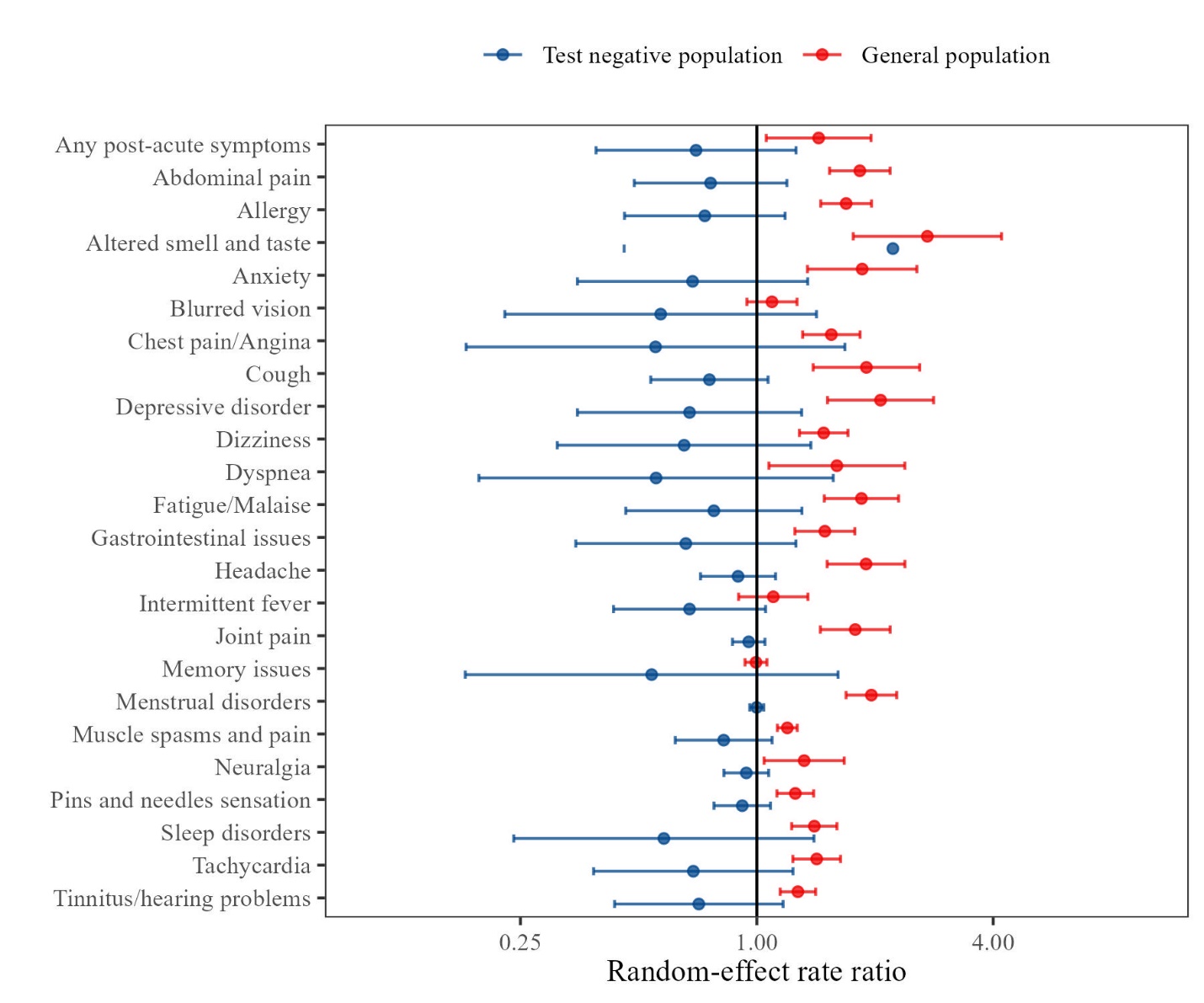


## **Figure S7 Crude incidence rate ratio of post-acute COVID-19 symptoms between the infected, test negative and general cohorts for the primary care databases.**


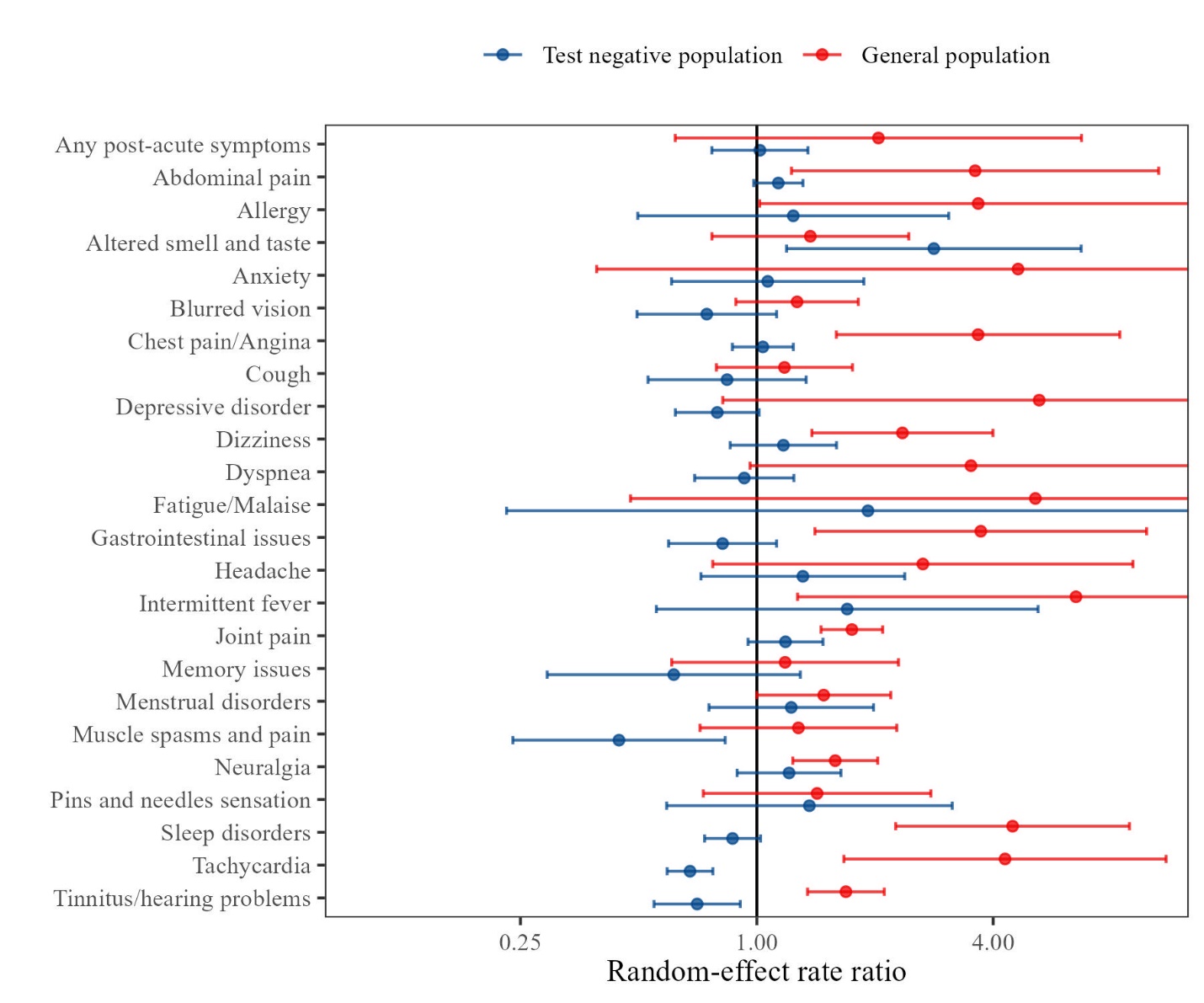


## **Figure S8 Crude incidence rate ratio of post-acute COVID-19 symptoms between the infected, test negative and general cohorts for the secondary care databases.**


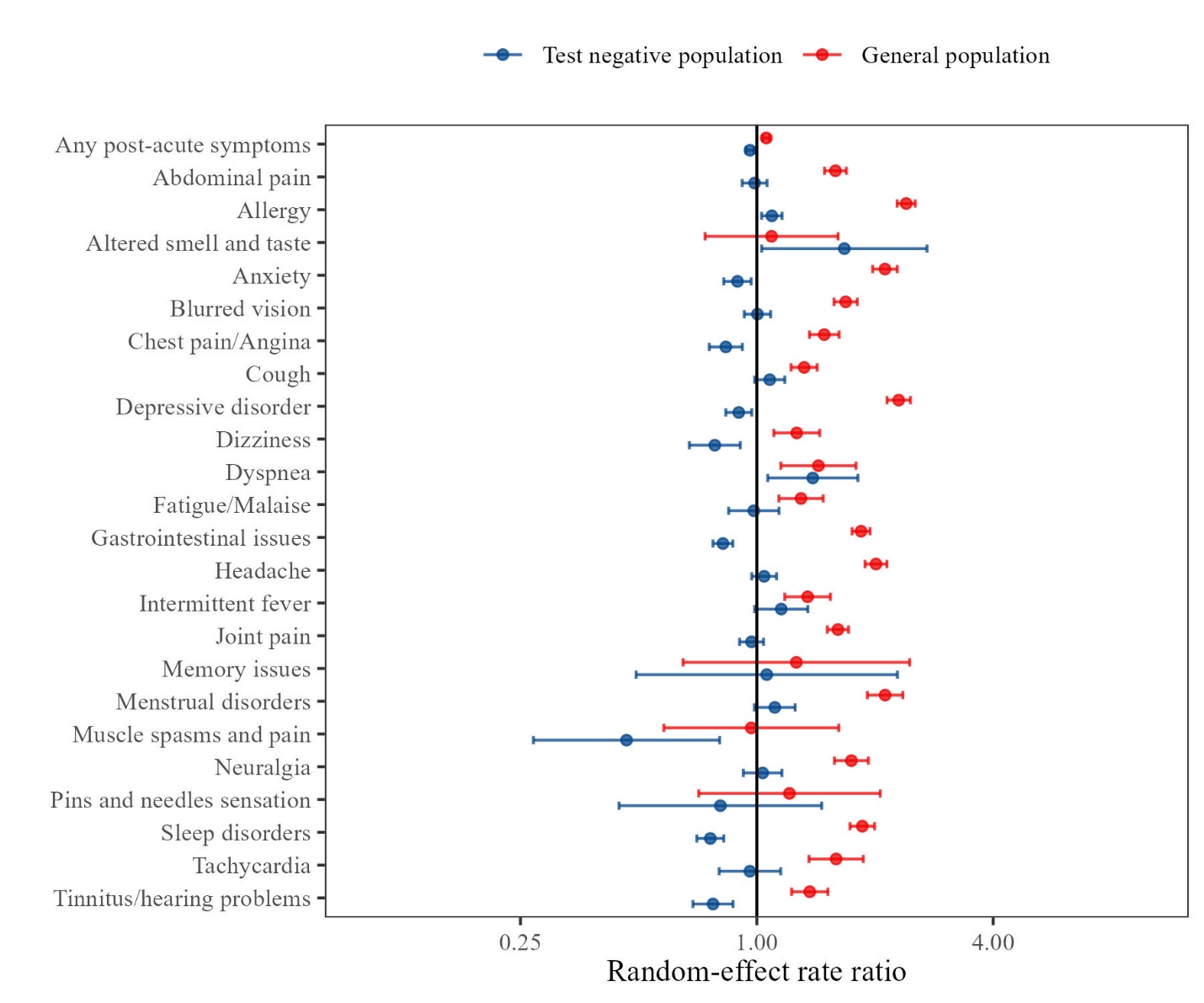


## **Figure S9 Crude incidence rate ratio of post-acute COVID-19 symptoms between the infected, test negative and general cohorts for the claims databases.**


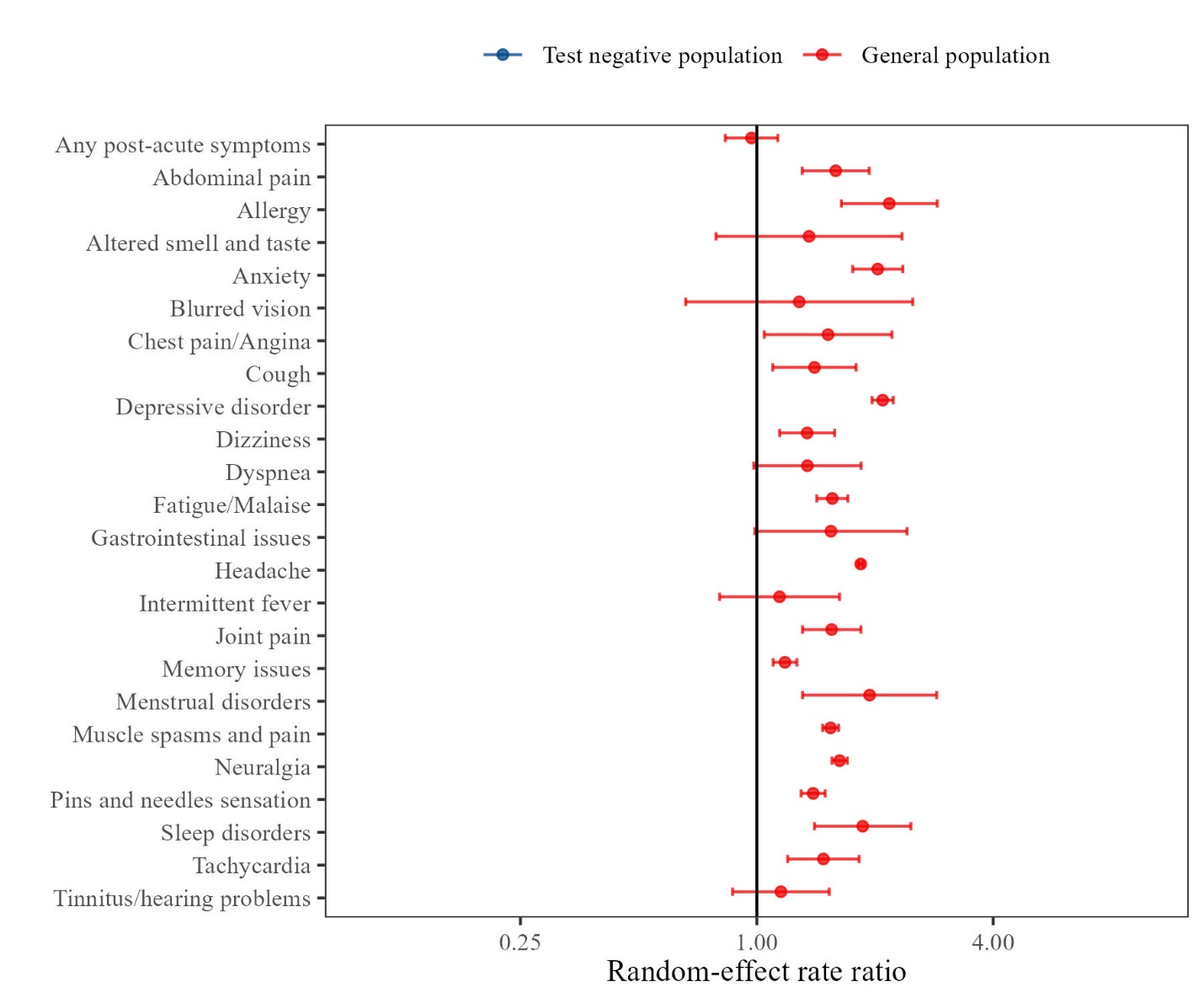


## **Figure S10 Crude incidence rate ratio of post-acute COVID-19 symptoms between the infected, test negative and general cohorts for the registries databases.**


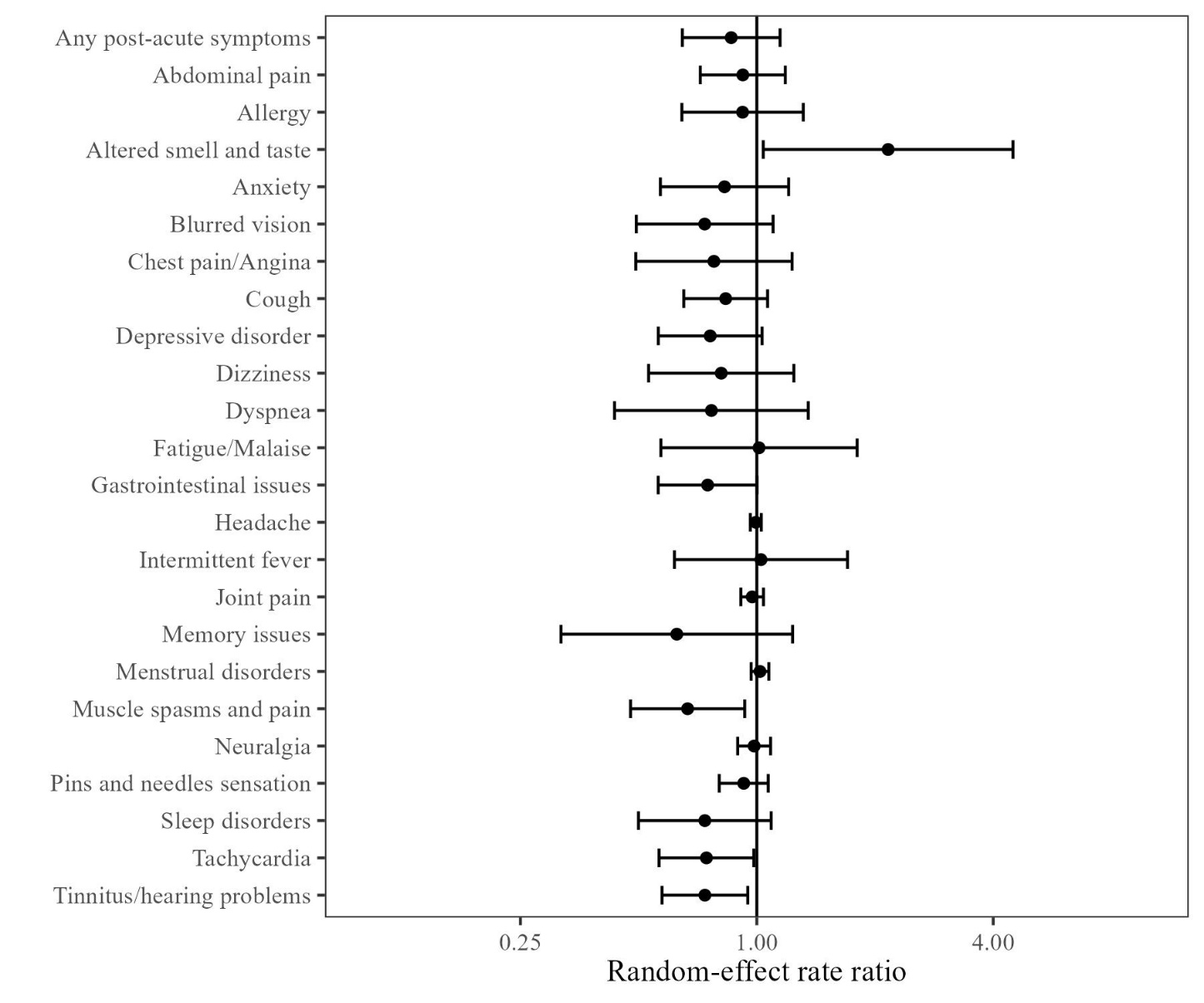


## **Figure S11 Crude incidence rate ratio of post-acute COVID-19 symptoms between the infected cohort and test negative cohort.**

**Attrition figures**

**
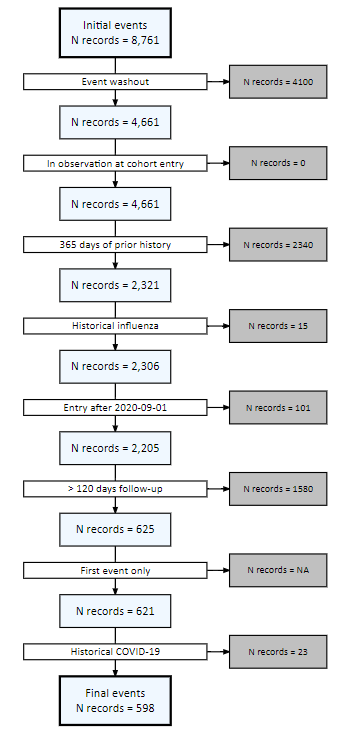
**
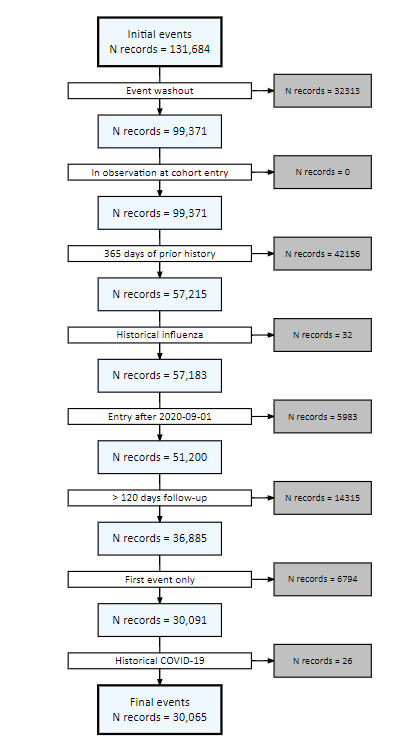


Figure S12: Attrition for the infected cohort (left) and test negative cohort (right) for the database AUSOM.


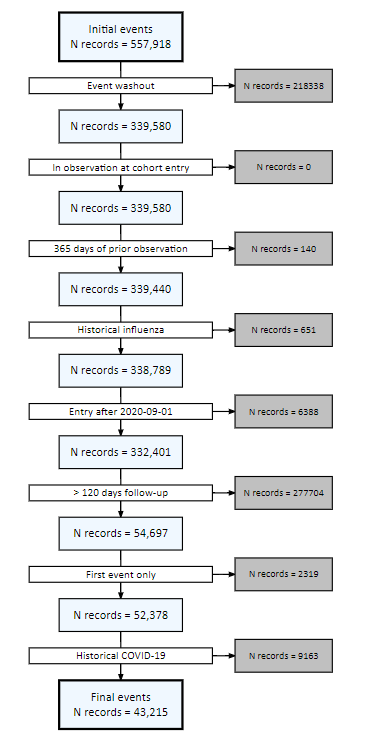

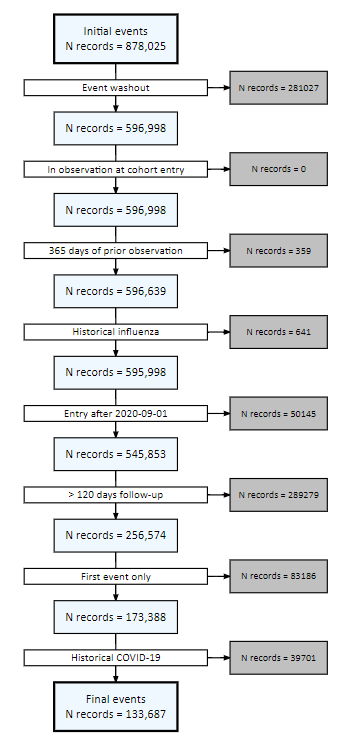


Figure S13: Attrition for the infected cohort (left) and test negative cohort (right) for the database CORIVA.


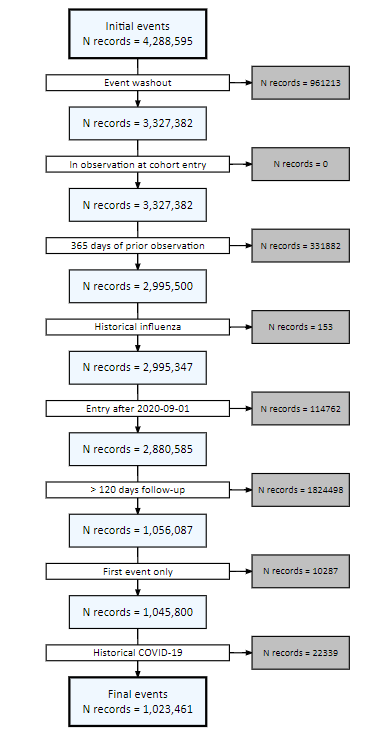

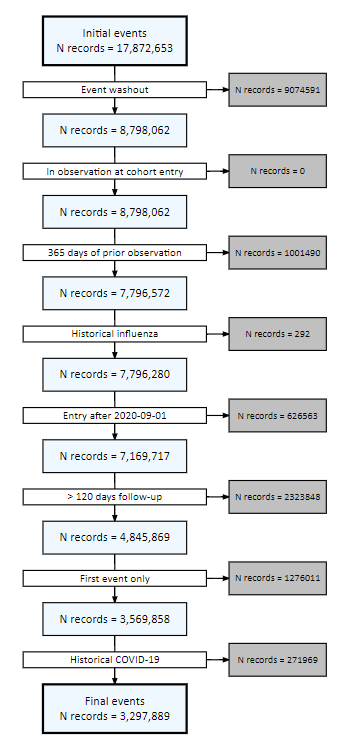


Figure S14: Attrition for the infected cohort (left) and test negative cohort (right) for the database CPRDAurum.


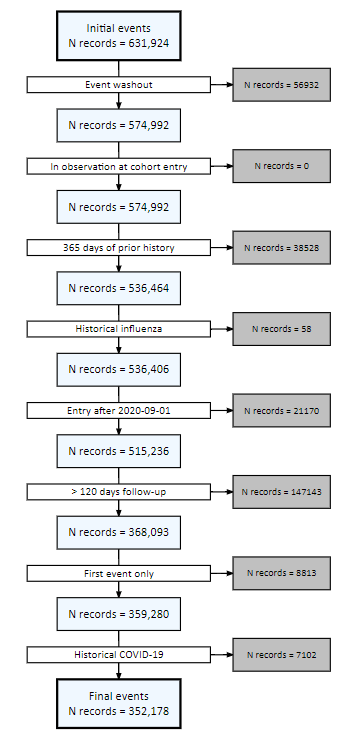

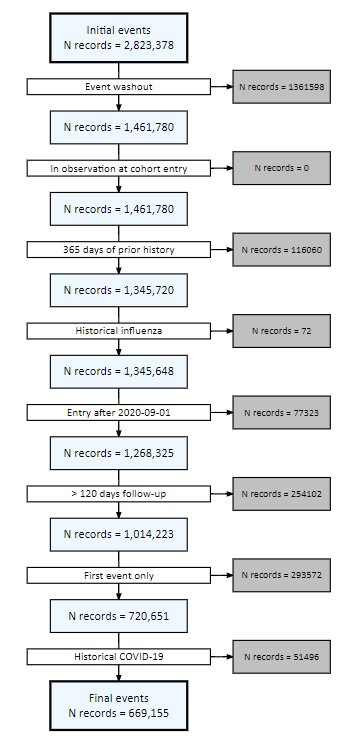


Figure S15: Attrition for the infected cohort (left) and test negative cohort (right) for the database CPRDGold.


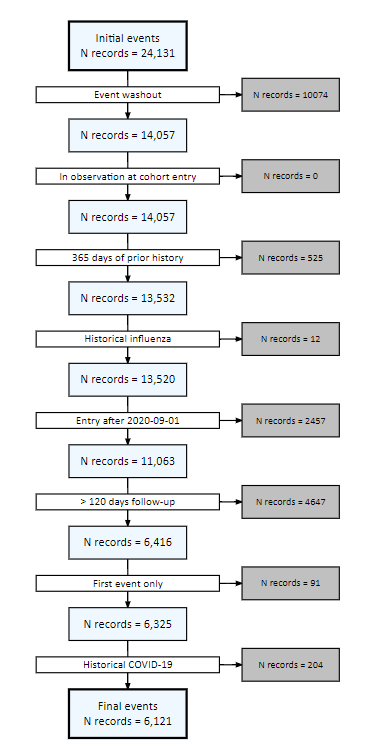

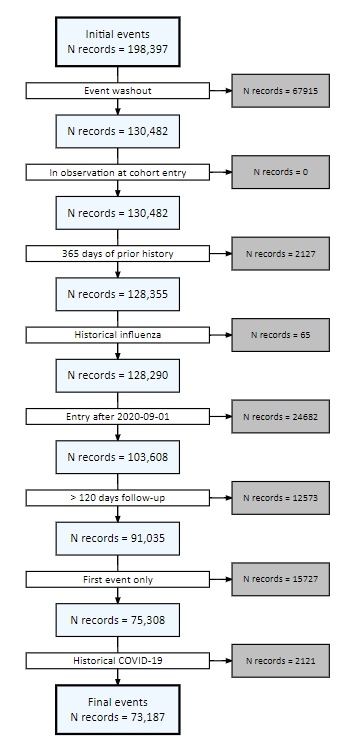


Figure S16: Attrition for the infected cohort (left) and test negative cohort (right) for the database eDOL.


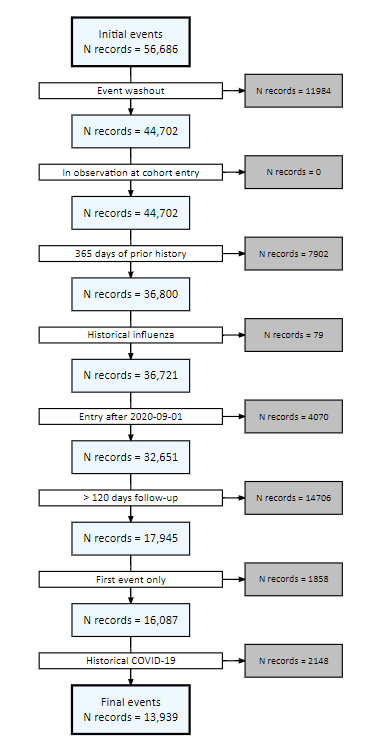

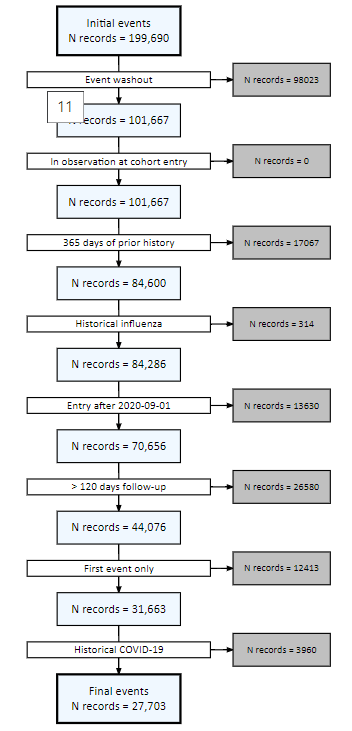


Figure S17: Attrition for the infected cohort (left) and test negative cohort (right) for the database IMASIS.


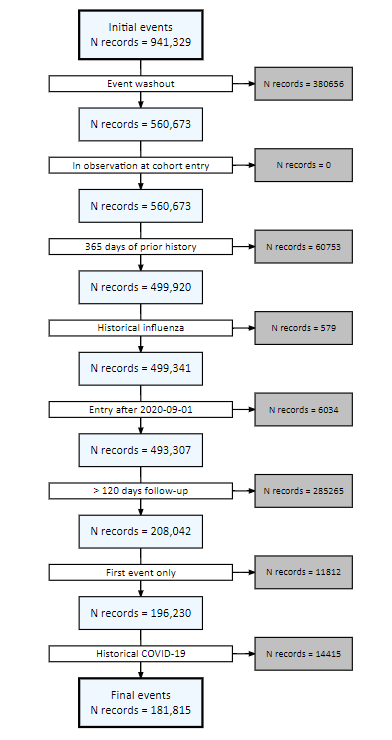

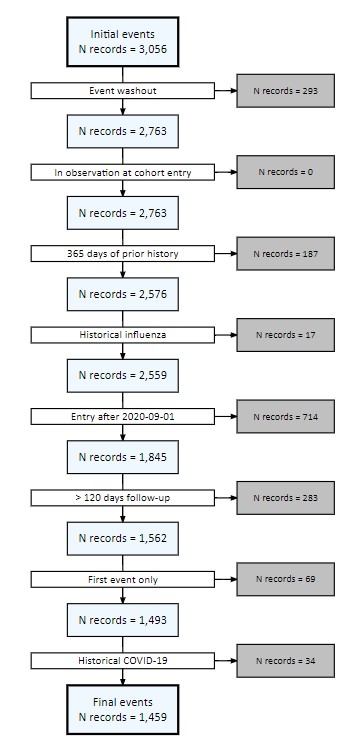


Figure S18: Attrition for the infected cohort (left) and test negative cohort (right) for the database IPCI.


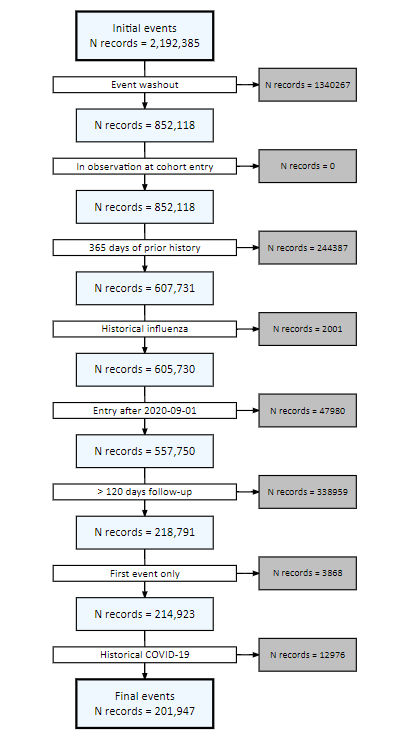


Figure S19: Attrition for the infected cohort for the database Pharmetrics.


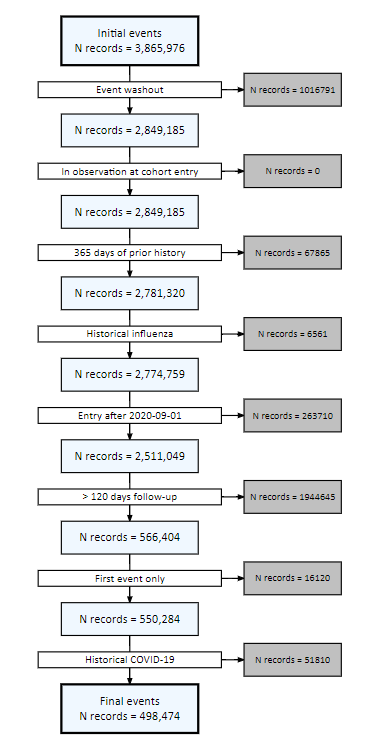


Figure S20: Attrition for the infected cohort for the database SIDIAP.


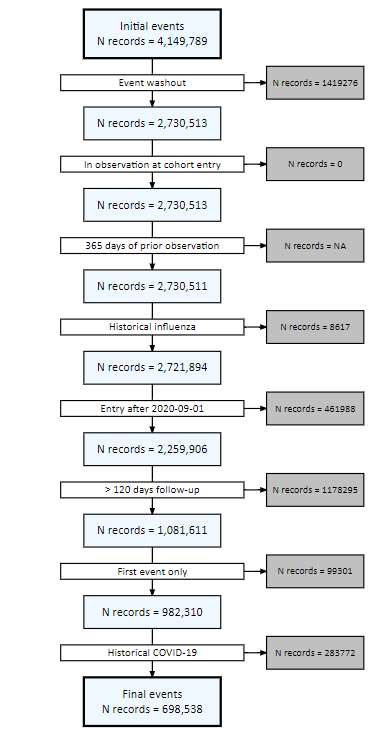


Figure S21: Attrition for the infected cohort for the database NLHR@UiO.

## Table S1. Counts for the main denominators and numerators of the incidence calculations in all databases.

| **Database name** | **Number of people infected** | **Ratio of PACS events** | **Number of people with a negative test** | **Ratio of PACS events** | **Number of people in the general population** | **Ratio of PACS events** |
| --- | --- | --- | --- | --- | --- | --- |
| AUSOM | 598 | 17.56 | 30065 | 16.18 | 298415 | 65.82 |
| CORIVA | 43215 | 22.27 | 133687 | 21.07 | 246965 | 101.92 |
| CPRD GOLD | 352178 | 8.81 | 669155 | 12.72 | 1201790 | 17.66 |
| CPRD Aurum | 1023461 | 14.54 | 3297889 | 14.54 | 4875478 | 45.56 |
| EDOL | 6121 | 2.81 | 73187 | 3.3 | 1799447 | 1.49 |
| IMASIS | 13939 | 13.48 | 27703 | 15.98 | 98931 | 36.79 |
| IPCI | 181815 | 19.54 | 1459 | 42.84 | 604882 | 38.14 |
| PharMetrics Plus | 201947 | 39.26 | NA | NA | 4314348 | 252.66 |
| SIDIAP | 498474 | 21.3 | NA | NA | 1768470 | 30.64 |
| NLHR@UiO | 698538 | 19.37 | NA | NA | 2517546 | 50.09 |

Note: The columns displaying ratio of PACS events are calculated as n_PACS_events_in_cohort / n_people_in_cohort. That is why some of them are above 100%.

## Table S2. Characterisation of the infected cohort, test negative cohort and general population in all databases

| **Database** | **Cohort** | **N** | **Sex female** | **Age** | **Index date** |
| --- | --- | --- | --- | --- | --- |
| AUSOM | infection | 598 | 321 (53.68 %) | 50 [29,68] | 2022-05-22 [2022-08-05,2022-05-09] |
| AUSOM | test negative | 30065 | 15953 (53.06 %) | 55 [38,68] | 2021-07-27 [2021-12-06,2021-04-04] |
| AUSOM | general population | 398114 | 201403 (50.59 %) | 49 [33,61] | 2020-09-01 [2020-09-01,2020-09-01] |
| CORIVA | infection | 43215 | 23351 (54.03 %) | 35 [20,51] | 2021-02-05 [2021-03-18,2020-12-18] |
| CORIVA | test negative | 133687 | 71422 (53.42 %) | 39 [25,55] | 2021-01-26 [2021-05-14,2020-11-10] |
| CORIVA | general population | 438269 | 228349 (52.1 %) | 41 [25,59] | 2020-09-01 [2020-09-01,2020-09-01] |
| CPRD Aurum | infection | 1023461 | 538899 (52.65 %) | 33 [17,49] | 2021-07-10 [2021-09-21,2021-01-04] |
| CPRD Aurum | test negative | 3297889 | 1782808 (54.06 %) | 35 [18,52] | 2021-01-29 [2021-07-13,2020-11-19] |
| CPRD Aurum | general population | 13709413 | 6835656 (49.86 %) | 38 [21,57] | 2020-09-01 [2020-09-01,2020-09-01] |
| CPRD GOLD | infection | 352178 | 191578 (54.4 %) | 35 [19,51] | 2021-10-10 [2021-12-27,2021-06-22] |
| CPRD GOLD | test negative | 669155 | 371319 (55.49 %) | 38 [20,54] | 2021-05-31 [2021-09-23,2020-12-13] |
| CPRD GOLD | general population | 3597966 | 1809553 (50.29 %) | 41 [23,59] | 2020-09-01 [2020-09-01,2020-09-01] |
| IMASIS | infection | 13939 | 7300 (52.37 %) | 58 [42,72] | 2021-09-28 [2022-04-04,2021-04-13] |
| IMASIS | test negative | 27703 | 15088 (54.46 %) | 60 [41,75] | 2021-07-28 [2022-01-20,2021-03-02] |
| IMASIS | general population | 166450 | 86523 (51.98 %) | 49 [33,67] | 2020-09-01 [2020-09-01,2020-09-01] |
| IPCI | infection | 181815 | 97711 (53.74 %) | 39 [21,55] | 2021-12-20 [2022-02-14,2021-03-03] |
| IPCI | test negative | 1459 | 895 (61.34 %) | 58 [39,74] | 2020-12-07 [2021-03-03,2020-10-14] |
| IPCI | general population | 1533819 | 781452 (50.95 %) | 42 [22,60] | 2020-09-01 [2020-09-01,2020-09-01] |
| Pharmetrics | infection | 201947 | 105983 (52.48 %) | 43 [27,59] | 2021-03-25 [2021-09-14,2020-12-24] |
| Pharmetrics | test negative | NA | NA | NA | NA |
| Pharmetrics | general population | 8244168 | 4275098 (51.77 %) | 47 [27,65] | 2020-09-01 [2020-12-31,2020-09-01] |
| SIDIAP | infection | 498474 | 267415 (53.65 %) | 34 [18,51] | 2021-02-11 [2021-07-11,2020-12-01] |
| SIDIAP | test negative | NA | NA | NA | NA |
| SIDIAP | general population | 6025038 | 3047181 (50.58 %) | 43 [24,59] | 2020-09-01 [2020-09-01,2020-09-01] |
| UiO | infection | 698538 | 349625 (50.05 %) | 33 [19,50] | 2020-12-24 [2021-04-01,2020-10-22] |
| UiO | test negative | NA | NA | NA | NA |
| UiO | general population | 5550904 | 2746672 (49.48 %) | 39 [21,58] | 2020-09-01 [2020-09-01,2020-09-01] |
| eDOL_CHUM | infection | 6121 | 3444 (56.27 %) | 49 [30,71] | 2021-09-28 [2022-02-15,2021-03-21] |
| eDOL_CHUM | test negative | 73187 | 40668 (55.57 %) | 43 [28,66] | 2021-07-02 [2022-01-03,2021-01-18] |
| eDOL_CHUM | general population | 1910472 | 988147 (51.72 %) | 42 [25,64] | 2020-09-01 [2020-09-01,2020-09-01] |

Note: counts are reported for the total number of subjects (N) and for sex (female). Percentage is also reported for sex. Median and IQR are reported for age and index date variables.

## Table S3. Latest data availability for all databases

| **Database name** | **Latest data availability** |
| --- | --- |
| AUSOM | 28-02-2023 |
| CORIVA | 28-02-2022 |
| CPRD GOLD | 30-06-2022 |
| CPRD Aurum | 31-03-2022 |
| EDOL | 31-12-2022 |
| IMASIS | 31-12-2022 |
| IPCI | 31-12-2022 |
| PharMetrics Plus | 30-06-2022 |
| SIDIAP | 31-03-2022 |
| NLHR@UiO | 31-12-2021 |
